# Antibiofilm and cytotoxic metabolites from the entomopathogenic fungus *Samsoniella aurantia*

**DOI:** 10.3762/bjoc.21.23

**Published:** 2025-02-11

**Authors:** Rita Toshe, Syeda J Khalid, Blondelle Matio Kemkuignou, Esteban Charria-Girón, Paul Eckhardt, Birthe Sandargo, Kunlapat Nuchthien, J Jennifer Luangsa-ard, Till Opatz, Hedda Schrey, Sherif S Ebada, Marc Stadler

**Affiliations:** 1 Department of Microbial Drugs, Helmholtz Centre for Infection Research GmbH (HZI) and German Centre for Infection Research (DZIF), Inhoffenstrasse 7, 38124 Braunschweig, Germany; 2 Institute of Microbiology, Technische Universität Braunschweig, Spielmannstrasse 7, 38106 Braunschweig, Germany; 3 Johannes Gutenberg University Mainz, Department of Chemistry, 55128 Mainz, Germany; 4 National Center for Genetic Engineering and Biotechnology (BIOTEC), National Science and Technology Development Agency (NSTDA), 113 Thailand Science Park, Phahonyothin Rd., Khlong Nueng, Khlong Luang, Pathum Thani 12120, Thailand; 5 Department of Pharmacognosy, Faculty of Pharmacy, Ain Shams University, Cairo 11566, Egypt

**Keywords:** antibiofilm, antiproliferative, beauvericin, insect pathogen, *Samsoniella*

## Abstract

During the course of our studies on the secondary metabolism of rare, hitherto untapped Thai insect-associated fungi, the ethyl acetate (EtOAc) extract derived from solid-state cultivation of *Samsoniella aurantia* on rice afforded one previously undescribed tetramic acid derivative, farinosone D (**1**), along with the known 2-pyridones, farinosones A (**2**) and B (**3**), and the known cyclodepsipeptides beauvericins A–C (**4**–**6**). All isolated compounds were assessed for their antimicrobial and cytotoxic activities while farinosones D (**1**) and A (**2**) were selected for biofilm inhibitory activity assay. Farinosone B (**3**) and beauvericins A–C (**4**–**6**) showed significant cytotoxic activities with IC_50_ values in the low micromolar to nanomolar range against several mammalian cell lines. On the other hand, farinosone A (**2**), which lacked potent cytotoxic effects, revealed potent antibiofilm activity, inhibiting approximately 70% of *Staphylococcus aureus* biofilms at concentrations as low as 3.9 µg/mL.

## Introduction

A microbial biofilm represents an assemblage of planktonic cells to overcome hostile living conditions on either biotic or abiotic surfaces. Structurally, biofilms are polymicrobial consortia embedded in an extracellular polymeric substance (EPS) that plays a pivotal role in surface adhesion, enhancement of gene exchange, antimicrobial resistance, and protection against host immune and inflammatory responses [1]. Biofilm-forming microbes were found to be up to 1,000 times more resistant to antibiotics than their planktonic forms [2]. According to the fact sheets of the National Institute of Health (NIH) and the Center for Disease Control and Prevention (CDC), 65 and 80% of all microbial and chronic infections are associated with biofilm formation, respectively, including serious infections affecting mucosal membranes or on implants yielding serious threats in hospitalized patients [3–4]. Consequently, biofilm-based antibiotic resistance poses a major threat to human health and substantially exacerbates healthcare and economic burdens, thereby prompting the urgent need for novel therapeutic strategies and antibiofilm agents.

During our ongoing research targeting potential antibiofilm metabolites from fungi, we explored entomopathogenic species such as those belonging to the genera *Beauveria* and *Metarhizium* that are known as biocontrol agents against insect pests like mosquitoes and ticks in agricultural and forestry applications [5]. Secondary metabolites produced by entomopathogenic fungi have garnered attention due to their diverse biological activities, encompassing antimicrobial, antiviral, and anticancer properties [6]. With concerns about biofilm formation and resistance escalating in the medical field, entomopathogenic fungi have emerged as a promising source of novel bioactive compounds [5]. Our recent exploration of *B. neobassiana* highlighted this growing interest, affording one tetramic acid and five 2-pyridone derivatives that revealed potential antibiofilm activity against *Staphylococcus aureus* biofilms [7]. These results encouraged us to explore a member of the Cordycipitaceae, in the genus *Samsoniella* that was segregated from *Akanthomyces* based on morphological and molecular evidence [6], which has to the best of our knowledge not been studied before on its secondary metabolism.

The current study deals with the isolation and characterization of the major bioactive principles of the ex-holotype strain of *Samsoniella aurantia*.

## Results and Discussion

### Structure elucidation

**Figure 1:**
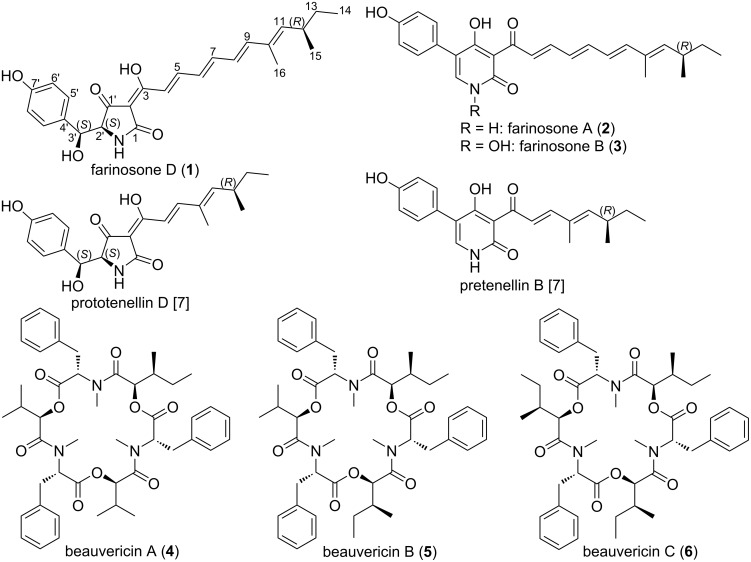
Chemical structures of compounds **1**–**6**, prototenellin D and pretenellin B [7].

Compound **1** (Figure 1) was purified as a yellow amorphous solid. The molecular formula of **1** was determined as C_25_H_29_NO_5_ indicating twelve degrees of unsaturation based on the HRESIMS that revealed a protonated molecule at *m*/*z* 424.2126 [M + H]^+^ (calculated 424.2118) and a sodium adduct at *m*/*z* 446.1947 [M + Na]^+^ (calculated 446.1962). The UV–vis spectrum of **1** (Figure S3, Supporting Information File 1) revealed a prominent absorption peak (λ_max_) at 434 nm in the visible region reflected by being yellow-colored and suggesting the presence of an extended conjugated π-system in its structure.

**Figure 2:**
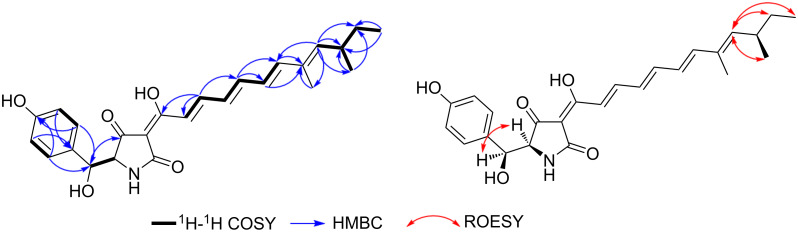
Key ^1^H-^1^H COSY, HMBC and ROESY correlations of **1**.

The ^1^H NMR and ^1^H–^1^H COSY spectra of **1** (Table 1, Figure 2, Figure S7, Supporting Information File 1) confirmed this feature by revealing one spin system extending over six olefinic protons starting from a proton peak at δ_H_ 6.58 (d, *J* = 15.0 Hz, H-4) to a proton resonance at δ_H_ 7.39 (dd, *J* = 15.0, 11.5 Hz, H-5) indicating their existence in *E* configuration. Then, the spin system extends over two overlapping olefinic protons at δ_H_ 6.89 (H-6)/6.90 (H-7) proceeding to two olefinic protons at δ_H_ 6.38 (dd, *J* = 15.0, 11.0 Hz, H-8) and 6.56 (d, *J* = 15.0 Hz, H-9). In addition, the ^1^H–^1^H COSY spectrum of **1** (Figure 2) revealed another characteristic spin system between two pairs of electromagnetically equivalent aromatic protons at δ_H_ 6.60 (d, *J* = 8.4 Hz, H-6'/8') and δ_H_ 7.01 (d, *J* = 8.4 Hz, H-5'/9') suggesting the presence of a 1,4-disubstituted aromatic ring in **1**. A literature search of **1** based on the deduced structural features revealed that it is a related derivative to two yellow pyridone pigments, farinosones A and B, that were previously reported from *Cordyceps farinosa* syn. *Paecilomyces farinosus* [8–9].

**Table 1:** ^1^H and ^13^C NMR data of farinosone D (**1**).

pos.	δ_C_,^a,b^ type	δ_H_^a^ (multi, *J* [Hz])	pos.	δ_C_,^a,b^ type	δ_H_^a^ (multi, *J* [Hz])

1	175.5, CO		14	11.9, CH_3_	0.80 (t, 7.4)
2	100.6, C		15	20.4, CH_3_	0.94 (d, 6.6)
3	172.0, C		16	12.4, CH_3_	1.77 (d, 1.2)
4	130.0, CH	6.58 (d, 15.0)	1'	192.9, CO	
5	144.2, CH	7.39 (dd, 15.0, 11.5)	2'	67.8, CH	4.16 (br s)
6	119.4, CH	6.89 (overlapping)	3'	72.6, CH	4.87 (d, 3.1)
7	144.7, CH	6.90 (overlapping)	4'	130.0, C	
8	126.4, CH	6.38 (dd, 15.0, 11.0)	5',9'	128.3, CH	7.01 (d, 8.4, 2H)
9	143.9, CH	6.56 (d, 15.0)	6',8'	114.2, CH	6.60 (d, 8.4, 2H)
10	133.1, C		7'	156.5, C	
11	143.9, CH	5.51 (d, 9.6)	3'-OH		5.63 (br s)
12	34.2, CH	2.43 (m)	7'-OH		9.25 (br s)
13	29.7, CH_2_	α 1.25 (m) β 1.36 (m)			

^a^Measured in DMSO-*d*_6_ at 125 MHz for ^13^C and 500 MHz for ^1^H. ^b^Assigned based on HMBC and HSQC spectra.

A detailed comparison of the ^1^H and ^13^C NMR data of **1** and farinosones A/B revealed that instead of a deshielded pyridone aromatic proton at δ_H_ 7.54/8.14 ppm [8], compound **1** exhibited two aliphatic methine proton signals at δ_H_ 4.16 (br s, H-2') and 4.87 (d, *J* = 3.1 Hz, H-3') consisting of a common spin system in the ^1^H–^1^H COSY spectrum (Figure 2, Figure S7, Supporting Information File 1) and were directly correlated via HSQC spectrum to two sp^3^ carbon atoms at δ_C_ 67.8 (C-2') and 72.6 (C-3'), respectively. These results suggested that compound **1** possesses a tetramic acid moiety similar to prototenellin D (Figure 1), a precursor in the biosynthesis of tenellin [10], a 2-pyridone derivative purified from the entomopathogenic fungus *Beauveria bassiana* [11].

**Figure 3:**
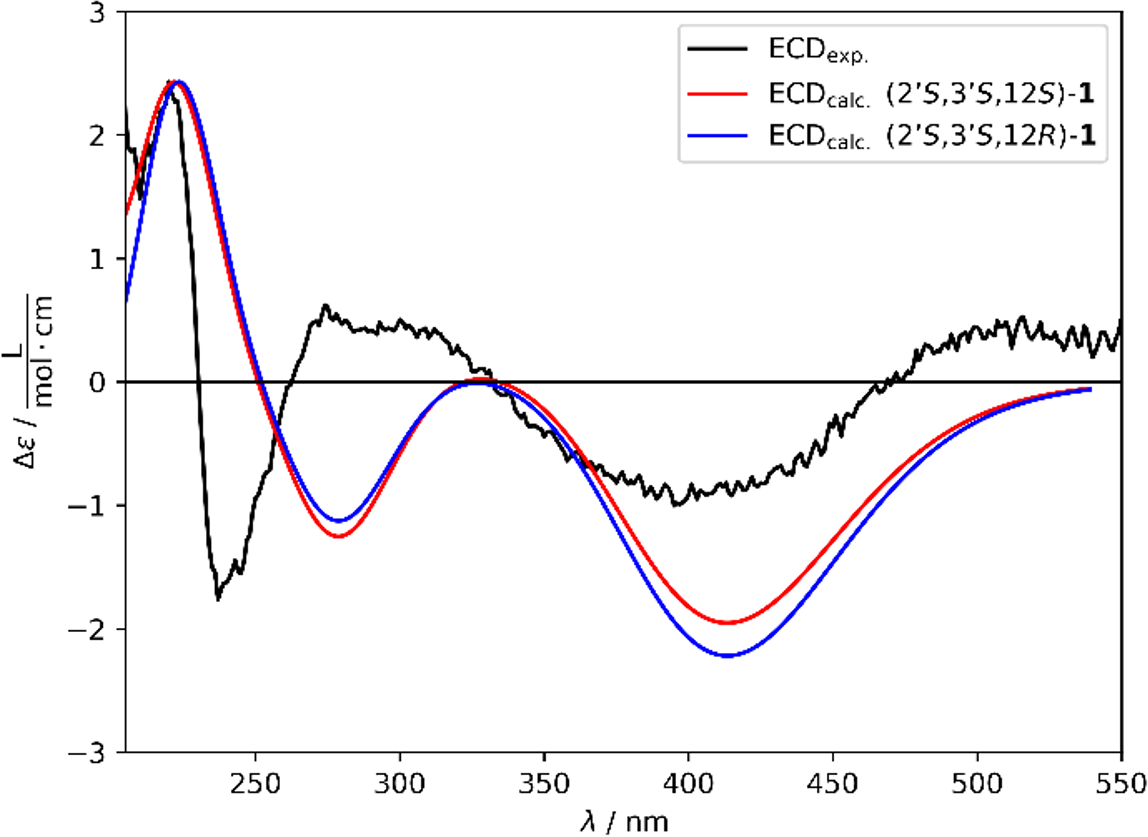
Comparison of experimental (black) and simulated Boltzmann-averaged (red: (2’*S*,3’*S*,12*S*)-**1**; green: (2’*S*,3’*S*,12*R*)-**1**) ECD spectra of compound **1**.

Further confirmation of the depicted structure of **1** was obtained via its HMBC spectrum (Figure 2, Figure S8, Supporting Information File 1) that revealed key correlations from the two aromatic protons H-5'/H-9' to the C-3' and in turn a key correlation for H-3' to the ketocarbonyl carbon at δ_C_ 192.9 (C-1') confirming the presence of a hydroxy group at C-3' and to be as depicted in Figure 1. Further key HMBC correlations were recognized including those from an olefinic proton at δ_H_ 5.51 (d, *J* = 9.6 Hz, H-11) to one allylic methyl group at δ_H_ 1.77 (d, *J* = 1.2 Hz, H_3_-16) and one doublet methyl group at δ_H_ 0.94 (d, *J* = 6.6 Hz, H_3_-15) indicating its presence in the middle between both methyl groups. The latter together with a terminal triplet methyl group at δ_H_ 0.80 (t, *J* = 7.4 Hz, H_3_-14) further revealed key correlation to one tertiary and one secondary sp^3^ carbon atoms at δ_C_ 34.2 (C-12) and 29.7 (C-13), respectively. The HMBC correlations (Figure 3) indicated that the side chain in **1** ends as –C(CH_3_)=CH-CH(CH_3_)-CH_2_-CH_3_ resembling those from farinosones A/B [8] and tenellin derivatives [10–11]. The relative configuration of **1** was elucidated based on its ROESY spectrum (Figure 2, Figure S10, Supporting Information File 1) that revealed key ROE correlations from H-2’ and H-3’ indicating their cofacial orientation whereas 3’-OH and 2’-NH are facing the opposite side of the molecule. In addition, further key ROE correlations were distinguished from H-11 to two methyl groups ascribed to H_3_-14 and H_3_-15 together with a diastereotopic methylene group at δ_H_ 1.25/1.36 (H_2_-13) confirming the depicted end of the aliphatic sidechain as –C(CH_3_)=CH-CH(CH_3_)-CH_2_-CH_3_ similar to that in farinosones A/B [8] and tenellin derivatives [10–11].

To establish the absolute configuration of **1**, an ECD spectrum was measured and compared to a simulated Boltzmann-averaged spectrum (Figure S11, Supporting Information File 1). The similarity factor for each stereoisomer was calculated (Table S1, Supporting Information File 1) [12]. The two diastereomers displaying the highest similarity factor between experimental and calculated spectrum are (2’*S*,3’*S*,12*S*) and (2’*S*,3’*S*,12*R*) (Figure 3) differing only at stereocenter C-12. As seen in Figure 3 and Table S1 (Supporting Information File 1), a differentiation between these two diastereomers based on the ECD spectra and similarity factor is not possible. However, the structural resemblance and the common biosynthetic origin with tenellin derivatives suggested that C-12 in **1** adopts (*R*) configuration as previously determined for prototenellin D [13]. Based on the aforementioned results, compound **1** was identified as a previously undescribed tetramic acid derivative named farinosone D.

In addition, five known secondary metabolites were identified based on HRESIMS and detailed 1D/2D NMR spectroscopic analyses along with comparison with the reported literature. The isolated compounds were differentiated into two 2-pyridone derivatives farinosones A (**2**) and B (**3**) [8] together with three cyclodepsipeptides beauvericins A–C (**4**–**6**) [14–15].

Farinosones (**1**–**3**) belong to a class of tetramic acid metabolites related to tenellin congeners that are prone to oxidative ring expansion affording their corresponding 2-pyridone derivatives. Based on a previously reported study investigating the biosynthesis of tenellin from *Beauveria bassiana* CBS110.25 [13], it was established that tenellin and a vast array of related metabolites could be synthesized through a cascade involving hybrid highly reducing polyketide synthase–non-ribosomal peptide synthetase (PKS–NRPS) [13].

**Figure 4:**
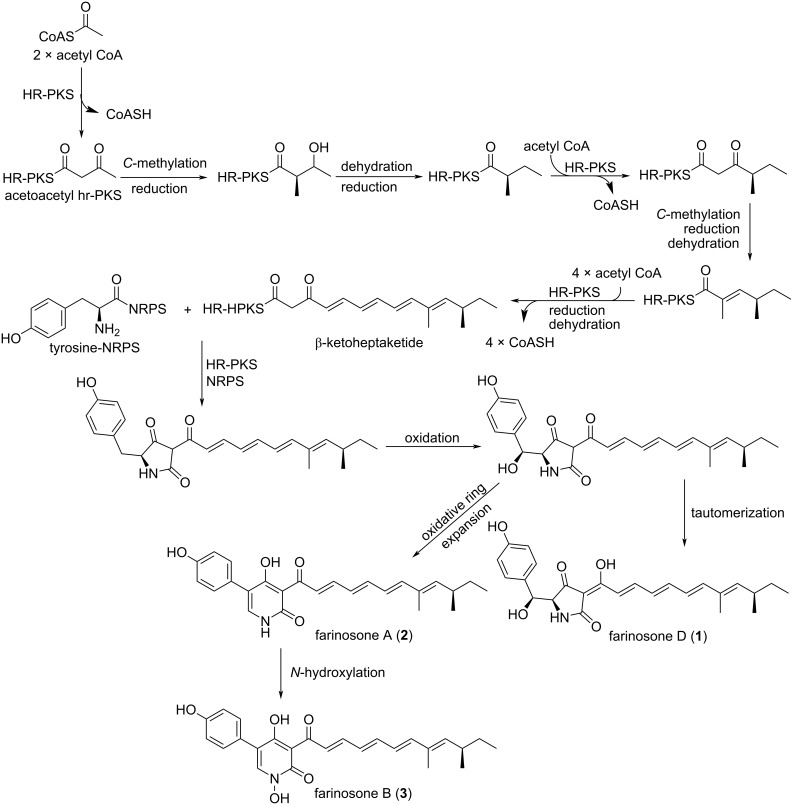
A plausible biosynthetic pathway of **1**–**3**.

Based on the structural relations between farinosones and tenellins differing only in the side chain length/the number of conjugated double bonds, farinosones were also postulated to be synthesized adopting a similar biosynthetic pathway. The biosynthesis of farinosones (Figure 4) begins with an acetate moiety that is extended six times by HR-PKS, programmed *C*-methylations took place after the first and second extensions with a cycle of full reduction occurring after the first extension. Subsequently, four acetate extensions with programmed reductions and dehydrations to afford β-ketoheptaketide that would in turn combine to a tyrosine moiety bound to NRPS to afford the first tetramic acid derivative. Then, oxygenation at C-2’ of the afforded first product followed by tautomerization would yield farinosone D (**1**), whereas oxidative ring expansion instead of tautomerization would produce the first 2-pyridone derivative, farinosone A (**2**) that through *N*-hydroxylation would reveal farinosone B (**3**).

### Biological evaluation

All the isolated compounds were assessed for their cytotoxic activity against a panel of seven different cancer cell lines. The results (Table 2) revealed that farinosone B (**3**) and beauvericins A–C (**4**–**6**) possess potent significant cytotoxic activities against all tested cell lines with IC_50_ values ranging from 0.2 to 6.2 µM, whereas farinosones D (**1**) and A (**2**) revealed weak to no cytotoxic activity. These results suggested that compounds **3**–**6** could be considered as potential cytotoxic molecules that might require further mechanistic investigation. In the antimicrobial activity assay (Table 2), farinosone B (**3**), beauvericins A (**4**) and B (**5**) were the most potent metabolites, especially against *S. aureus* and *Bacillus subtilis* with MIC values between 4.2 and 16.6 µg/mL. Intriguingly, farinosones D (**1**) and A (**2**) did not exhibit significant antimicrobial activity other than weak to moderate effects against *S. aureus* and *B. subtilis*. Therefore, **1** and **2** were selected as optimal candidates to assess their antibiofilm capacity against *S. aureus*. The results from biofilm assays demonstrated significant activity for both compounds, particularly compound **2** (Figure 2).

**Table 2:** Cytotoxicity (IC_50_) and antimicrobial activity (MIC) of **1**–**6**.

	IC_50_ (µM)						positive control
	
test cell line	**1**	**2**	**3**	**4**	**5**	**6**	epothilone B (nM)

Mouse fibroblast (L929)	n.a.	n.a.	0.6	1.2	1.0	0.8	0.65
Human endocervival adenocarcinoma (KB3.1)	47.3	79.0	0.5	1.2	1.0	0.8	0.17
Human prostate carcinoma (PC-3)	68.6	n.d.	6.2	n.d.	2.3	2.8	0.09
Human breast adenocarcinoma (MCF-7)	9.7	n.d.	0.2	n.d.	1.2	1.1	0.07
Human ovarian cancer (SKOV-3)	26.0	n.d.	0.3	n.d.	1.5	1.5	0.09
Human epidermoid carcinoma (A431)	18.2	n.d.	0.2	n.d.	1.0	1.0	0.06
Human lung carcinoma (A549)	52.0	n.d.	0.3	n.d.	3.0	3.2	0.05

test microorganism	MIC (µg/mL)						positive control (µg/mL)

*Staphylococcus aureus* (DSM 346)	66.6	33.3	16.6	16.6	8.3	n.i.	0.21^G^
*Escherichia coli* (DSM 1116)	n.i.	n.i.	66.6	n.i.	n.i.	n.i.	0.42^G^
*Bacillus subtilis* (DSM 10)	66.6	33.3	16.6	8.3	4.2	n.i.	16.6^O^
*Pseudomonas aeruginosa* (PA14)	n.i.	n.i.	n.i.	n.i.	n.i.	n.i.	0.21^G^
*Wickerhamomyces anomalus* (DSM 6766)	n.i.	n.d.	n.i.	n.d.	n.d.	n.i.	16.6^N^
*Candida albicans* (DSM 1665)	n.i.	n.i.	n.i.	n.i.	n.i.	n.i.	8.3^N^
*Acinetobacter baumannii* (DSM 30008)	n.i.	n.d.	n.i.	n.d.	n.d.	n.i.	0.52^C^
*Chromobacterium violaceum* (DSM 30191)	n.i.	n.d.	n.i.	n.d.	n.d.	n.i.	1.70^G^
*Schizosaccharomyces pombe* (DSM 70572)	n.i.	n.d.	66.6	n.d.	n.d.	n.i.	8.30^N^
*Mucor hiemalis* (DSM 2656)	n.i.	n.i.	n.i.	n.i.	n.i.	n.i.	8.30^N^
*Rhodotorula glutinis* (DSM 10134)	n.i.	n.d.	n.i.	n.d.	n.d.	n.i.	4.20^N^
*Mycolicibacterium smegmatis* (ATCC 700084)	n.i.	n.i.	n.i.	16.6	8.3	n.i.	1.70^K^

n.a.: no activity; n.i.: no inhibition up to 67 µg/mL; n.d.: not determined; G: gentamicin; O: oxytetracycline; N: nystatin; C: ciprofloxacin; K: kanamycin.

Based on our recent study, we reported antibiofilm activity of prototenellin D and pretenellin B (Table 3), isolated from the entomopathogenic fungus *Beauveria neobassiana* [7], against the formation of *S. aureus* biofilms. Structurally, prototenellin D and pretenellin B (Figure 1) comprise a tetramic acid entity and a 2-pyridone moiety, sharing the presence of similar aliphatic side chains that are two conjugated double bonds shorter than in farinosones D (**1**) and A (**2**), respectively.

**Table 3:** Inhibition of biofilm formation of *S. aureus* by **1** and **2**.

*Staphylococcus aureus* DSM 1104 biofilm inhibition (% ± SD)						

tested compound	conc. (µg/mL)					
	
	125	62.5	31.25	15.62	7.8	3.9

farinosone D (**1**)	69 ± 9	75 ± 9	78 ± 12	74 ± 11	32 ± 39	–
farinosone A (**2**)	72 ± 8	76 ± 12	78 ± 11	74 ± 11	76 ± 8	68 ± 13
prototenellin D [8]	84 ± 17	83 ± 9	80 ± 10	56 ± 8	53 ± 7	–
pretenellin B [8]	83 ± 6	83 ± 6	79 ± 8	52 ± 6	–	–

microporenic acid A (MAA)	78 ± 9	79 ± 8	78 ± 12	69 ± 11	–	

**Figure 5:**
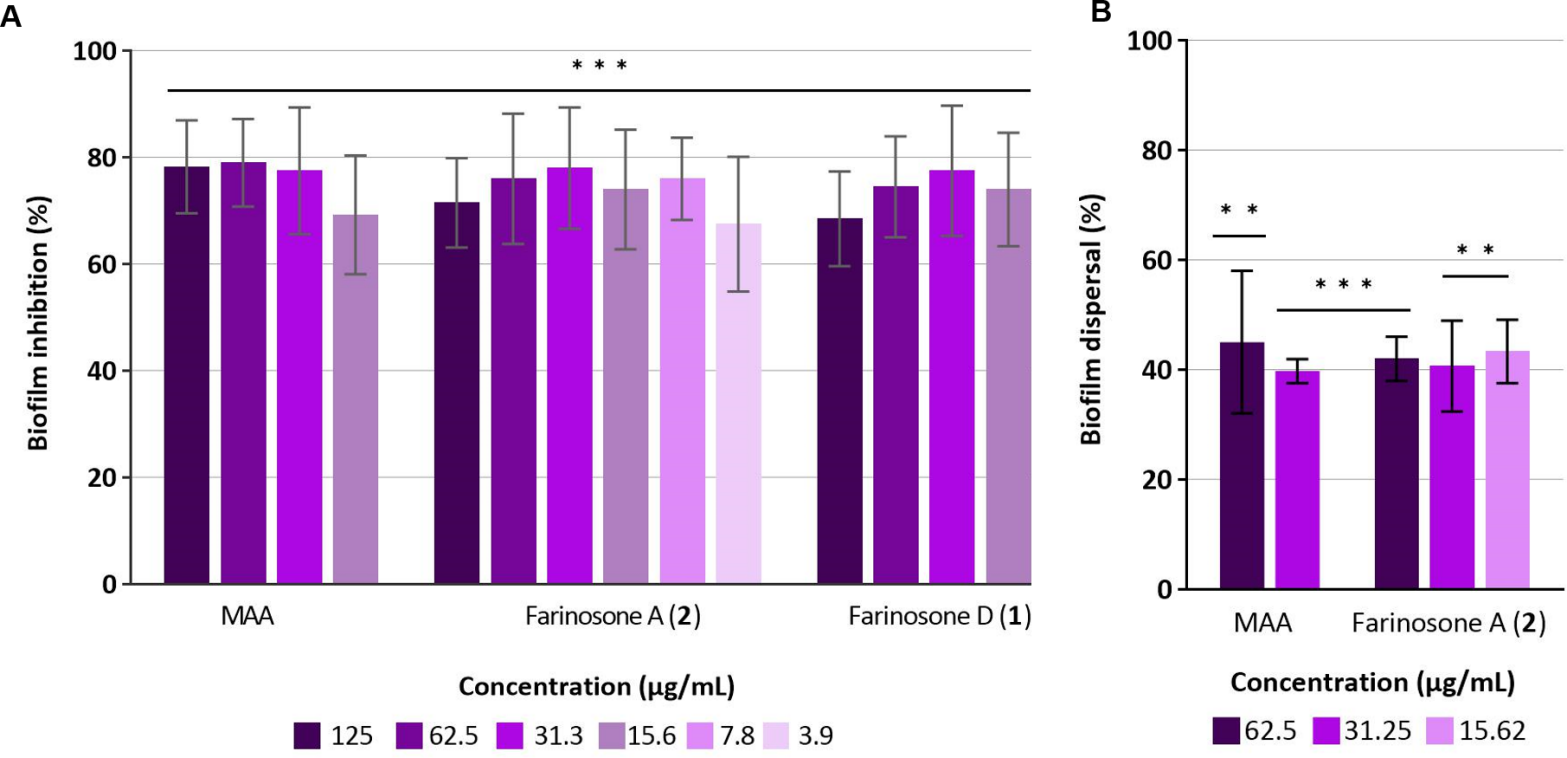
Biofilm inhibition and eradication assessment via CV staining assay. A) *S. aureus* biofilm inhibition by farinosone D (**1**) and farinosone A (**2**). B) *S. aureus* 24 hour biofilm dispersal by farinosone A (**2**). Microporenic acid A (MAA) was used as a positive control. Methanol was used as a solvent control and taken as 100%. Error bars indicate standard deviation (SD) of duplicates in two biological repeats; *p* values: * *p* < 0.05, ** *p* < 0.01, *** *p* < 0.001.

Therefore, in this study, supplementary screening assays were conducted to explore their antibiofilm properties. According to the crystal violet (CV) assay, both compounds exhibited significant activity in the *S. aureus* biofilm inhibition assay. Farinosone A (**2**) (Figure 5A) displayed activity at a concentration as low as 3.9 µg/mL, resulting in approximately 68% inhibition of biofilm formation. Similarly, farinosone D (**1**) demonstrated activity up to a concentration of 15.62 µg/mL, achieving a comparable inhibitory effect of approximately 74% at this dilution.

In addition, only farinosone A (**2**) (Figure 5B) demonstrated moderate activity in the preformed biofilm assay, dispersing a 24 hour preformed biofilm up to a concentration of 15.62 µg/mL with an eradication capacity of 43%.

**Figure 6:**
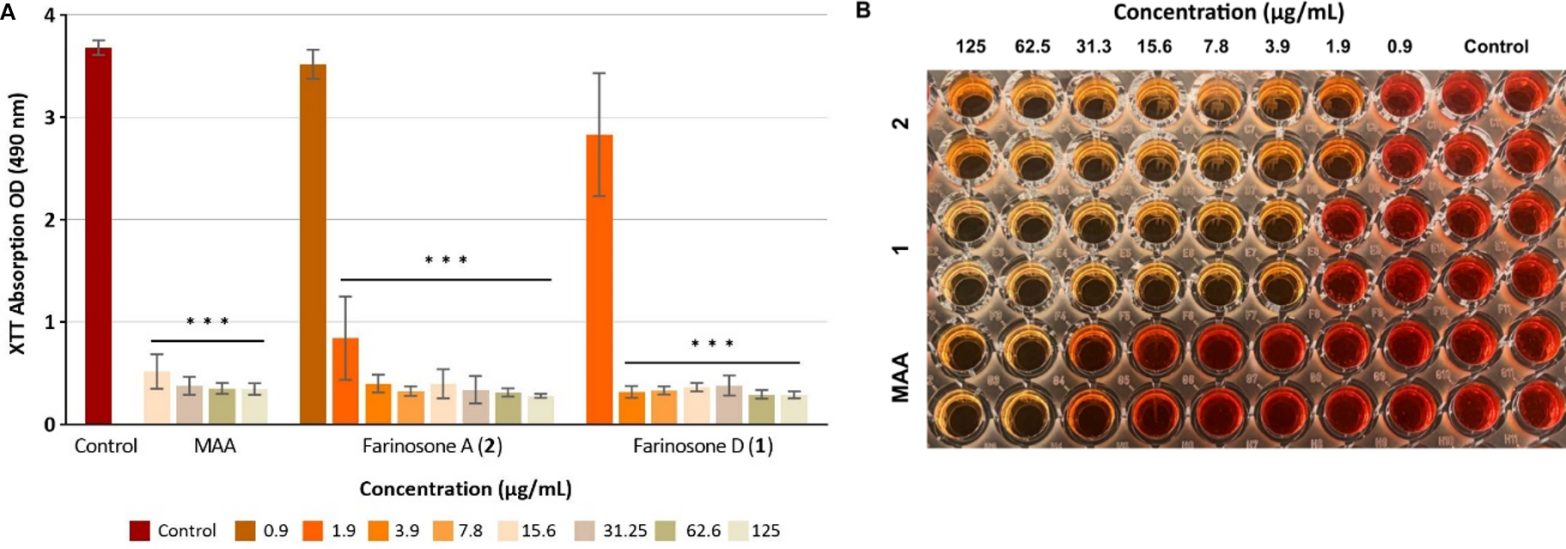
A) Metabolic activity in biomass of *S. aureus* biofilm treated with farinosones D (**1**) or A (**2**). Error bars indicate standard deviation of duplicates in two biological repeats (*n* = 4); *p* values: * *p* < 0.05, ** *p* < 0.01, *** *p* < 0.001. B) XTT microplate containing *S. aureus* biofilm treated with farinosones D (**1**) and A (**2**)**,** microporenic acid A (MAA) as a positive control and methanol as a solvent control and taken as 100%.

To evaluate whether the remaining biofilm biomass in the wells was metabolically active or not, a cell viability (XTT) assay was conducted after 24 hour treatment of *S. aureus* biofilms with farinosones D (**1**) and A (**2**). This distinction is pivotal in the evaluation of compounds for their antibiofilm efficacy, considering the potential scenario wherein a compound may affect the growth dynamics of cells without substantially disrupting the biofilm architecture or dispersing the encapsulated cells [16]. The results (Figure 6) yielded striking significance, revealing that the biomass remained metabolically inactive in biofilms treated with **1** and **2** low to concentrations of 3.9 and 1.9 µg/mL, respectively. It is worth noting here that both compounds followed a similar activity trend, reflecting the results from CV biofilm inhibition assay, where compound **2** was found to be more effective than compound **1**.

The genetic and molecular basis of biofilm formation in staphylococci are complex. This process involves at least two key properties: planktonic cell adherence to surfaces and the subsequent accumulation to form multilayered cell clusters [17]. To investigate the action of farinosones D (**1**) and A (**2**) on planktonic cells and their adherence to the substrate, growth curve and fibrinogen binding assay were conducted. The findings obtained from these assays demonstrated the efficacy of **1** and **2** in inhibiting the initial proliferation of planktonic cells, with inhibitory effects observed up to concentrations of 33.3 and 16.6 µg/mL in Mueller–Hinton broth (MHB) medium, 15.62 and 7.8 µg/mL in CASO medium, respectively (Figures S2A, S2B, S1A and S1B, Supporting Information File 1). Additionally, in fibrinogen binding assay, both compounds reduced the initial attachment of planktonic cells by approximately 30% at a concentration of 62.5 µg/mL (Figure S1C, Supporting Information File 1).

Based on the results of fibrinogen binding assay (Figure S1C, Supporting Information File 1), it is likely that the activity of farinosones D (**1**) and A (**2**) does not interfere with the factors essential for fibronectin binding, particularly the fibronectin-binding proteins such as fibronectin binding protein (FnBPA and B) [18]. FnBPs are adhesions and are known to play a key role in biofilm formation of *S. aureus* although there are, of course, other binding factors that contribute to the formation of biofilms [19]. Overall, the results suggested that farinosones D (**1**) and A (**2**) were effective against *S. aureus* biofilms as demonstrated by their ability to reduce biomass and cell viability.

## Conclusion

Among the isolated compounds, and based on the results of bioassays, farinosones D (**1**) and A (**2**) were selected as potential candidates for biofilm assays due to their minimal to weak cytotoxicity. Microbiological assays aimed at identifying potential targets within the *S. aureus* biofilm revealed that these compounds did not significantly inhibit cell adherence to the substrate in binding assay nor they showed profound dispersion of preformed biofilms in biofilm eradication assay in the low µg/mL range. However, they effectively reduced metabolic activity and cell viability at concentrations as low as 3.9 µg/mL for farinosone D (**1**) and 1.9 µg/mL for farinosone A (**2**), as demonstrated by the XTT assay. This reduction in metabolic activity may explain why *S. aureus* was unable to form robust biofilms, as reflected in the CV biofilm inhibition assay. Therefore, it can be reasonably concluded that farinosones D (**1**) and A (**2**) hold significant potential as biofilm inhibitors because of their ability to inhibit biofilm formation at very low concentrations, suggesting their value in early-stage therapeutic applications against *S. aureus* infections.

## Experimental

### General experimental methods

Optical rotations at 20 °C were measured with an Anton Paar MCP 150 circular polarimeter (Anton Paar, Graz, Austria). UV–vis spectra spanning the 190–600 nm range were acquired using a Shimadzu UV2450 spectrophotometer (Shimadzu, Kyoto, Japan). Electronic circular dichroism (ECD) spectra were obtained using a J-815 spectropolarimeter (JASCO Pfungstadt, Germany). The optical rotation, ECD, and UV–vis spectra of the newly described secondary metabolites were assessed in MeOH (Uvasol, Merck, Darmstadt, Germany).

Analytical HPLC chromatograms and electrospray ionization mass spectra (ESIMS) were acquired using a Thermo-Fischer Scientific UltiMate 3000 Series UPLC (Waltham, MA, USA) equipped with a C_18_ column (Acquity UPLC BEH 50 × 2.1 mm, 1.7 µm; Waters, Milford, MA, USA) and an amaZon speed ESI-Ion trap-MS (Bruker) with a sample injection volume of 2 µL and a flow rate of 0.6 mL/min. A gradient elution was applied using a mobile phase consisting of solvent A: H_2_O + 0.1% formic acid (v/v) and solvent B: acetonitrile (MeCN) + 0.1% formic acid (v/v). The gradient started at 5% B, gradually increasing to 100% B over 20 min, followed by a 10 min hold at 100% B, with UV–vis detection in the range from 190 to 600 nm.

High-resolution electrospray ionization mass spectra (HRESIMS) were obtained using an Agilent 1200 Infinity Series HPLC-UV system (Agilent Technologies, Santa Clara, CA, USA) equipped with a C_18_ Acquity UPLC BEH analytical column (50 × 2.1 mm, 1.7 µm; Waters, Milford, MA, USA) connected to a maXis^®^ Electrospray Time-Of-Flight mass spectrometer (ESI-TOF-MS; Bruker). The experimental conditions for acquiring the HRESIMS data were identical to those used for ESIMS.

### Fungal strain

The ex-holotype strain of *Samsoniella aurantia* BCC 55782 (corresponding type specimen BBH 33739) was isolated from a lepidopteran larva found on the leaf litter buried in the soil, on October 6, 2012.

### Cultivation and extraction

*Samsoniella aurantia* was cultivated on potato dextrose agar (PDA: 200 g potato, 20 g dextrose, 15 g agar in 1 L distilled water, Difco). Mycelial plugs, each with a diameter of 7.0 mm, were inoculated with rice in 10 × 1 L Erlenmeyer flasks, each containing 180 g of rice and 180 mL of distilled water that were foremost autoclaved, and the mixture was incubated for 16 days under static conditions at room temperature until full mycelial growth was achieved. Upon reaching optimal growth, the cultures underwent extraction. The mycelia were initially soaked in acetone overnight, followed by two rounds of ultrasonic extraction with freshly added acetone for each cycle. Resultant suspensions were filtered, and the liquid was collected. The combined liquid phases underwent vacuum evaporation at 40 °C, forming a semi-solid residue dispersed in water. Liquid–liquid extraction against EtOAc (1:1) was conducted twice. The organic phases were combined, filtered, and evaporated under vacuum, while the aqueous phase was discarded.

### Isolation of compounds **1**–**6**

The obtained organic crude extract (15 g) was analyzed by LC–MS. Thereafter, it was fractionated by vacuum liquid chromatography (VLC) using silica gel as a stationary phase. The elution procedure was performed using a chromatographic scheme of two different gradients as follows: 1) *n*-heptane/EtOAc (1:0, 7:3, 3:7, 0:1) followed by acetone/MeOH (9:1, 7:3, 1:1, 0:1) along with one last fraction of MeOH 100% using 0.1% formic acid. Nine fractions of 1 L each were eluted that were collected and evaporated separately under reduced pressure to dryness. For fractions 3, 4, 6 and 8 were further purified using a PLC 2250 preparative HPLC system (Gilson, Middleton, WI, USA). For fractions 3, 4, and 8, a Gemini C_18_ column (250 × 50 mm, 10 μm; Phenomenex, Aschaffenburg, Germany) was implemented, while for fraction 6, a Nucleodur C_18_ HTec column (250 × 21 mm, 10 μm; Macherey-Nagel, Düren, Germany) served as the stationary phase. The mobile phase consisted of deionized water (H_2_O) + 0.1% formic acid as solvent A and MeCN + 0.1% formic acid as solvent B. The flow rate was set at 30 mL/min for fractions 3, 4, and 8, and 20 mL/min for fraction 6. The collected fraction volume for each run was 15 mL.

In the purification of fraction 3 (1,315 mg), a gradient elution was implemented, starting from 60% B and progressing to 100% B in 30 minutes. The gradient was then held at 100% B for 10 minutes, resulting in the isolation of **6** (30 mg, *t*_R_ = 34 min). For the purification of fraction 4 (306 mg), a gradient elution was applied, transitioning solvent B from 30% to 100% over 85 minutes. Subsequently, it was maintained at 100% for 15 minutes. This purification protocol led to the isolation of **1** (9.1 mg, *t*_R_ = 67 min), **3** (19.5 mg, *t*_R_ = 81 min), and **5** (2.8 mg, *t*_R_ = 89 min). For fraction 6 (489 mg), a gradient elution was applied starting from 50% B and progressing to 100% B in 28 minutes. The gradient was then kept at 100% B for 5 minutes, resulting in the isolation of **2** (1.2 mg, *t*_R_ = 19 min). Fraction 8 (269 mg) underwent a purification process involving a gradient elution from 15% to 100% solvent B over 75 minutes. The gradient was then held at 100% B for 10 minutes, leading to the isolation of **4** (21.4 mg, *t*_R_ = 66 min).

Farinosone D (**1**): Yellow amorphous solid; 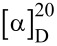 –264 (*c* 0.1, MeOH); UV–vis (MeOH) λ_max_ (log ε): 371.5 (4.09), 292 (3.69), 224.5 (3.91), 221 (3.91), 201 (4.14) nm; ECD (*c* = 0.059 mM; MeOH) λ [nm], (Δε) 500 (0.5), 400 (−1.0), 300 (0.5), 265 (0.6) 218 (2.4); NMR data (^1^H: 500 MHz, ^13^C: 125 MHz, DMSO-*d*_6_) see Table 1; HRESIMS *m*/*z*: [M – H_2_O + H]^+^ calcd for C_25_H_28_NO_4_^+^, 406.2026; found, 406.2020; [M + H]^+^ calcd for C_25_H_30_NO_5_^+^, 424.2118; found, 424.2126; [M + Na]^+^ calcd for C_25_H_29_NNaO_5_^+^, 446.1962; found, 446.1947.

Farinosone A (**2**): Pale yellow amorphous solid; UV–vis (MeOH) λ_max_: 368, 224, 200 nm; NMR data (^1^H: 500 MHz, ^13^C: 125 MHz, acetone-*d*_6_) comparable to the previously described spectral data [8]; HRESIMS *m*/*z*: [M + H]^+^ calcd for C_25_H_28_NO_4_^+^, 406.2103; found, 406.2103.

Farinosone B (**3**): Bright yellow powder; UV–vis (MeOH) λ_max_: 346, 230 nm; NMR data (^1^H: 500 MHz, acetone-*d*_6_) comparable to the previously described spectral data [8]; HRESIMS *m*/*z*: [M – H_2_O + H]^+^ calcd for C_25_H_26_NO_4_^+^, 404.1856; found, 404.1855; [M + H]^+^ calcd for C_25_H_28_NO_5_^+^, 422.1962; found, 422.1962; [M + Na]^+^ calcd for C_25_H_27_NNaO_5_^+^, 444.1782; found, 444.1782.

Beauvericin A (**4**): Yellow solid powder; UV–vis (MeOH) λ_max_: 344, 221 nm; NMR data (^1^H: 500 MHz in DMSO-*d*_6_) comparable to the previously described spectral data [14–15]; HRESIMS *m/z*: [M + H]^+^ calcd for C_46_H_60_N_3_O_9_^+^, 798.4324; found, 798.4324; [M + NH_4_]^+^ calcd for C_46_H_63_N_4_O_9_^+^, 815.4590; found, 815.4588; [M + Na]^+^ calcd for C_46_H_59_N_3_NaO_9_^+^, 820.4144; found, 820.4142.

Beauvericin B (**5**): Yellow amorphous solid; UV–vis (MeOH) λ_max_: 344, 250, 210 nm; NMR data (^1^H: 500 MHz, DMSO-*d*_6_) comparable to the previously described spectral data [14–15]; HRESIMS *m*/*z*: [M + H]^+^ calcd for C_47_H_62_N_3_O_9_^+^, 812.4481; found, 812.4489; [M + NH_4_]^+^ calcd for C_47_H_65_N_4_O_9_^+^, 829.4746; found, 829.4743; [M + Na]^+^ calcd for C_47_H_61_N_3_NaO_9_^+^, 834.4300; found, 834.4301.

Beauvericin C (**6**): Yellow oil; UV–vis (MeOH) λ_max_: 342, 248, 210 nm; NMR data (^1^H: 500 MHz, DMSO-*d*_6_) comparable to the previously described spectral data [14–15]; HRESIMS *m*/*z*: [M + H]^+^ calcd for C_48_H_64_N_3_O_9_^+^, 826.4637; found, 826.4651; [M + NH_4_]^+^ calcd for C_48_H_67_N_4_O_9_^+^, 843.4903; found, 843.4903; [M + Na]^+^ calcd for C_48_H_63_N_3_NaO_9_^+^, 848.4457; found, 848.4459.

### Antimicrobial assays

All compounds isolated in this study underwent examination for their antimicrobial efficacy using a serial dilution assay spanning concentrations from 67 to 0.5 µg/mL. The experimental design followed established protocols [20–21]. The minimum inhibitory concentration (MIC) against a diverse spectrum of pathogens was determined, including five fungal species: *Candida albicans* (DSM 1665), *Mucor hiemalis* (DSM 2656), *Schizosaccharomyces pombe* (DSM 70572), *Rhodotorula glutinis* (DSM 10134), and *Wickerhamomyces anomalus* (DSM 6766). Additionally, various Gram-positive bacteria such as *Staphylococcus aureus* (DSM 346), *Bacillus subtilis* (DSM 10), *Mycobacterium smegmatis* (ATCC 700084), and Gram-negative bacteria including *Acinetobacter baumannii* (DSM 30008), *Escherichia coli* (DSM 1116), *Chromobacterium violaceum* (DSM 30191), and *Pseudomonas aeruginosa* (PA14) were included in the assessment. Gentamicin and nystatin served as positive controls against most bacteria and all fungi, respectively. For specific microorganisms (*A. baumannii, B. subtilis,* and *M. smegmatis*), ciprofloxacin, oxytetracycline, and kanamycin were used as positive controls.

### Cytotoxicity assays

The cytotoxic potential of the isolated compounds was determined using the MTT assay over a concentration range of 1 to 37 µg/mL against two mammalian cell lines: mouse fibroblasts (L-929) and human endocervical adenocarcinoma (KB-3.1) applying a previously described protocol [20–21]. Compounds exhibiting cytotoxic properties against these two cell lines were further assessed against prostate cancer (PC-3), breast cancer (MCF-7), ovarian cancer (SKOV-3), epidermoid carcinoma (A431) and lung cancer (A-549) cell lines. Epothilone B served as the positive control. Following a 5 day incubation period, the minimum concentration required to achieve 50% growth inhibition (IC_50_ values) was determined.

### Biofilm inhibition sssay

Biofilm inhibition activity of farinosones D (**1**) and A (**2**) was evaluated against *Staphylococcus aureus* (DSM 1104) according to the previously described protocol [22]. Briefly, *S. aureus* was cultured, adjusted to 0.001 McFarland standard, and incubated with serial dilutions of the compounds (125–0.9 µg/mL) in 96-well plates (TPP Tissue Culture ref no. 92196) for 24 hours. Biofilm inhibition was measured using crystal violet (CV) staining, with methanol as the control and microporenic acid A as the positive control [21]. Error bars indicate SD with duplicates in two biological repeats (*n* = 4).

### Biofilm dispersal assay against *Staphylococcus aureus*

To evaluate the biofilm dispersal activity of farinosones D (**1**) and A (**2**) on 24 hour old biofilms of *S. aureus* (DSM 1104), biofilms were first formed in 96-well plates (TPP Tissue Culture ref no. 92196) for 24 hours. After first incubation, supernatant was removed, fresh media and serial dilutions of farinosones D (**1**) and A (**2**) (125–0.9 µg/mL) were added and incubated for another 24 hours. Biofilm dispersal was measured using CV staining, with methanol as the solvent control and MAA as the positive control [21]. Error bars indicate SD with duplicates in two biological repeats (*n* = 4).

### Assessment of cell viability in treated *S. aureus* biofilms via XTT assay

Methoxynitrosulfophenyltetrazolium carboxanilide (XTT) assay was employed to evaluate cell viability in *S. aureus* (DSM 1104) biofilms treated with farinosones D (**1**) and A (**2**). After 24 hours’ incubation of treated biofilms, supernatant was discarded, wells were washed with PBS and 0.3 mg/mL final concentration of XTT (Cell Profile XTT kit, Roche) prepared in PBS was added (150 μL/well) [23]. Methanol was the solvent control and MAA was used as a positive control. Plates were incubated for 4 hours at 37 °C with shaking, and absorbance was measured at 490 nm (Synergy 2, BioTek). Error bars indicate SD with duplicates in two biological repeats (*n* = 4).

### Growth curve of *S. aureus*

The growth of *S. aureus* (DSM 1104) was studied in CASO with 4% glucose and Mueller–Hinton broth (MHB), following a previously described protocol with minor changes [23]. A fresh overnight culture of *S. aureus* (DSM 1104) at 0.001 McFarland standard was incubated for 24 hours with serial dilutions (125–0.9 µg/mL) of farinosones D (**1**) and A (**2**) in a 96-well microtiter plate (Greiner 96 Flat Transparent REF 82.1581001). The cultures were maintained at 37 °C with shaking at 510 rpm, and absorbance at 600 nm was measured every 30 minutes using a Spark multimode microplate reader (Tecan). Methanol (2.5%) served as the solvent control to confirm unhindered growth of *S. aureus* under the given conditions. Additionally, absorbance readings were taken for blank (only media) and media with compounds **1** and **2** to offset any false positive test results. The experiments were repeated twice with duplicates.

### Fibrinogen binding assay

The fibrinogen binding assay was carried out according to a recently published protocol, with minor adjustments [23–25]. An overnight culture of *S. aureus* (DSM 1104) was adjusted to an optical density comparable to 0.01 McFarland standard. Each 1 mL of this culture was combined with serial dilutions (125, 62.5, 31.25, 7.8, and 3.9 µg/mL) of test compounds in Eppendorf tubes (Eppendorf, Hamburg, Germany). After incubating for 7 hours at 37 °C and 120 rpm, bacterial cells were centrifuged, resuspended in PBS, and 100 µL of each suspension was added to wells of a 96-well microtiter plate (TPP tissue culture, ref. no 92196, TPP) coated with 20 µg/mL bovine fibrinogen (Sigma-Aldrich). Following incubation and washing, cells were fixed with 25% formaldehyde and assessed for biomass using CV staining. Methanol (2.5%) served as the solvent control, with MAA as the positive control [22]. Error bars indicate standard deviation among duplicates in two biological repeats (*n* = 4).

### Statistical analysis

The distinction between the samples and the control group was assessed through a two-tailed Student's *t*-test. The level of statistical significance was set at *p* < 0.05. The examination was conducted utilizing GraphPad Prism 9^®^ (GraphPad Software, San Diego, CA, USA).

### Computational details

The distribution of conformers was simulated using Spartan’10 [26] on the PM6 level of theory [27]. Density-functional theory (DFT) calculations were computed utilizing the Gaussian program package, revision C.01 [28]. The geometry of the conformers was optimized on the B3LYP/6-311+G(2d,p) [29–32] level of theory with tight cutoffs on forces and step size. The IEFPCM solvent model [33] was used to simulate the solvent effects of methanol and frequency calculations were used to determine the conformers as minima. Conformers 4.5 kcal/mol above the lowest energy conformer and double conformers were discarded. Time-dependent DFT calculations were carried out on the same level of theory. A Boltzmann-averaged spectra were created and compared to the experimental spectrum using SpecDis 1.71 [12].

## Supporting Information

File 1 HRESIMS profiles and NMR spectroscopic data of compounds **1**–**6**.

## Data Availability

All data that supports the findings of this study is available in the published article and/or the supporting information of this article.
