# Supplementary material for: Antibiofilm and cytotoxic metabolites from the entomopathogenic fungus Samsoniella aurantia
Source: Beilstein J Org Chem. 2025 Feb 11;21:327–39. doi: 10.3762/bjoc.21.23 (PMC11833177; doi:10.3762/bjoc.21.23)
Supplement: File 1 — HRESIMS profiles and NMR spectroscopic data of compounds 1–6. [file Beilstein_J_Org_Chem-21-327-s001.pdf]

BEILSTEIN JOURNAL OF ORGANIC CHEMISTRY

Supporting Information

for

Antibiofilm and cytotoxic metabolites from the entomopathogenic fungus *Samsoniella aurantia*

Rita Toshe, Syeda J. Khalid, Blondelle Matio Kemkuignou, Esteban Charria-Girón, Paul Eckhardt, Birthe Sandargo, Kunlapat Nuchthien, J. Jennifer Luangsa-ard, Till Opatz, Hedda Schrey, Sherif S. Ebada and Marc Stadler

Beilstein J. Org. Chem. **2025,** *21,* 327–339. doi:10.3762/bjoc.21.23

HRESIMS profiles and NMR spectroscopic data of compounds 1–6

License and Terms: This is a supporting information file under the terms of the Creative Commons Attribution License (https://creativecommons.org/licenses/by/4.0). Please note that the reuse, redistribution and reproduction in particular requires that the author(s) and source are credited and that individual graphics may be subject to special legal provisions.
The license is subject to the *Beilstein Journal of Organic Chemistry* terms and conditions: (https://www.beilstein-journals.org/bjoc/terms)

Contents of Supporting Information

#	Contents	Page
**1**	Biofilm inhibition assay	**S2**
**2**	Figure S1. A. Effect of farinosone D (**1**) on growth of *S. aureus* at varying concentrations in CASO medium. B. Effect of farinosone A (**2**) on growth of *S. aureus* at varying concentrations in CASO medium. C. Effect of farinosones D (**1**) and A (**2**) relative percent adhesion of *S. aureus* to fibrinogen.	**S3**
**3**	Figure S2. A. Effect of farinosone D (**1**) on growth of *S. aureus* at varying concentrations in Müller Hinton Broth (MHB) medium. B. Effect of farinosone A (**2**) on growth of *S. aureus* at varying concentrations in Müller Hinton Broth (MHB) medium.	**S3**
**4**	Figure S3. LRESIMS spectrum of **1**.	**S4**
**5**	Figure S4. HRESIMS spectrum of **1**.	**S5**
**6**	Figure S5. ^{1}H NMR spectrum of **1** in DMSO-d_6 at 500 MHz.	**S6**
**7**	Figure S6. ^{13}C NMR spectrum of **1** in DMSO-d_6 at 125 MHz.	**S7**
**8**	Figure S7. ^{1}H-^{1}H COSY spectrum of **1** in DMSO-d_6 at 500 MHz.	**S8**
**9**	Figure S8. HMBC spectrum of **1** in DMSO-d_6 at 500 MHz.	**S9**
**10**	Figure S9. HSQC spectrum of **1** in DMSO-d_6 at 500 MHz.	**S10**
**11**	Figure S10. ROESY spectrum of **1** in DMSO-d_6 at 500 MHz.	**S11**
**12**	Figure S11. Comparison of experimental (black) and simulated Boltzmann-averaged ECD spectra for compound **1**.	**S12**
**13**	Table S1. Similarity factor for each calculated stereoisomer of compound **1**.	**S12**
**14**	Figure S12. HRESIMS spectrum of **2**.	**S13**
**15**	Figure S13. ^{1}H NMR spectrum of **2** in acetone-d_6 at 500 MHz.	**S14**
**16**	Figure S14. DEPTQ spectrum of **2** in acetone-d_6 at 125 MHz.	**S15**
**17**	Figure S15. ^{1}H-^{1}H COSY spectrum of **2** in acetone-d_6 at 500 MHz.	**S16**
**18**	Figure S16. HMBC spectrum of **2** in acetone-d_6 at 500 MHz.	**S17**
**19**	Figure S17. HSQC spectrum of **2** in acetone-d_6 at 500 MHz.	**S18**
**20**	Figure S18. HRESIMS spectrum of **3**.	**S19**
**21**	Figure S19. ^{1}H NMR spectrum of **3** in acetone-d_6 at 500 MHz.	**S20**
**22**	Figure S20. ^{1}H-^{1}H COSY spectrum of **3** in acetone-d_6 at 500 MHz.	**S21**
**23**	Figure S21. HMBC spectrum of **3** in acetone-d_6 at 500 MHz.	**S22**
**24**	Figure S22. HSQC spectrum of **3** in acetone-d_6 at 500 MHz.	**S23**
**25**	Figure S23. HRESIMS spectrum of **4**.	**S24**
**26**	Figure S24. ^{1}H NMR spectrum of **4** in DMSO-d_6 at 500 MHz.	**S25**
**27**	Figure S25. ^{1}H-^{1}H COSY spectrum of **4** in DMSO-d_6 at 500 MHz.	**S26**
**28**	Figure S26. HMBC spectrum of **4** in DMSO-d_6 at 500 MHz.	**S27**
**29**	Figure S27. HSQC spectrum of **4** in DMSO-d_6 at 500 MHz.	**S28**
**30**	Figure S28. HRESIMS spectrum of **5**.	**S29**
**31**	Figure S29. ^{1}H NMR spectrum of **5** in DMSO-d_6 at 500 MHz.	**S30**
**32**	Figure S30. ^{1}H-^{1}H COSY spectrum of **5** in DMSO-d_6 at 500 MHz.	**S31**
**33**	Figure S31. HMBC spectrum of **5** in DMSO-d_6 at 500 MHz.	**S32**
**34**	Figure S32. HSQC spectrum of **5** in DMSO-d_6 at 500 MHz.	**S33**
**35**	Figure S33. HRESIMS spectrum of **6**.	**S34**
**36**	Figure S34. ^{1}H NMR spectrum of **6** in DMSO-d_6 at 500 MHz.	**S35**

Biofilm inhibition assay

Biofilm inhibition assays were conducted according to the methodology previously described (Soliga et al., 2021), targeting the inhibition of biofilm formation in *Staphylococcus aureus* (DSM 1104). *S. aureus* were cultured in CASO medium at 37 °C for 18 h, respectively, in a shaker at 120 rpm after being thawed from a −20 °C stock solution. The culture's optical density at 600 nm (OD600) was measured and adjusted to a 0.001 McFarland standard in CASO medium with 4% glucose for *S. aureus*, respectively. Microtiter plates were loaded with 150 µL of the bacterial suspension containing serially diluted test compounds (125–0.9 µg/mL) and incubated for 24 h at 37 °C. The evaluation of biofilm inhibition was performed by staining the biofilm with 0.1% crystal violet (CV). After staining, the plates were washed, and the biofilm dissolved in 95% ethanol. The absorbance of the dissolved biofilm was measured at 530 nm using a plate reader. The negative control was methanol (2.5%) while microporenic acid A at a concentration range of 7.8 to 125 µg/mL was used as the positive control for *S. aureus* (Chepkirui et al., 2018). All experiments were performed twice, and the standard deviation (SD) was maintained at or below 15% for *S. aureus*.

Differences between samples and the control group were determined by a two-tailed Student's *t*-test. Statistical significance was defined as $p < 0.05$. Analysis was carried out using GraphPad Prism 9® (GraphPad Software, San Diego, CA, USA).

References

Chepkirui, C.; Yuyama, KT.; Wanga, LA.; Decock, C.; Matasyoh, JC.; Abraham, WR.; Stadler, M. Microporenic acids A-G, biofilm inhibitors, and antimicrobial agents from the Basidiomycete *Microporus* Species. *J. Nat. Prod.* **2018**, 81, 778-784.

Soliga, KJ.; Bär, SI.; Oberhuber, N.; Zeng, H.; Schrey, H.; Schobert, R. Synthesis and bioactivity of ancorinoside B, A marine diglycosyl tetramic acid. *Mar. Drugs.* **2021**, 19, 583.

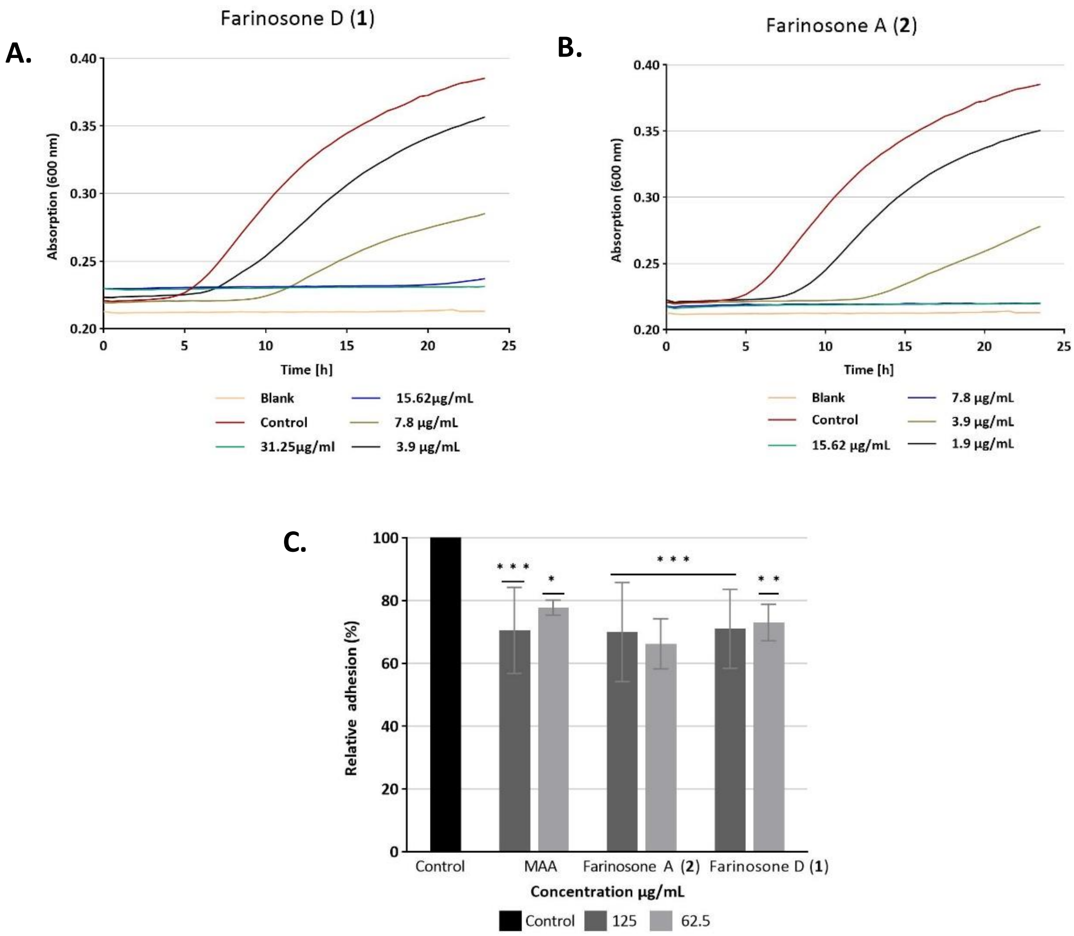

Figure S1. A. Effect of farinosone D (**1**) on growth of *S. aureus* at varying concentrations in CASO medium. B. Effect of farinosone A (**2**) on growth of *S. aureus* at varying concentrations in CASO medium. C. Effect of farinosones D (**1**) and A (**2**) relative percent adhesion of *S. aureus* to fibrinogen. Microporenic acid A (MAA) was used as positive control. Methanol was used as a solvent control and taken as 100%. Error bars indicate standard deviation of duplicates in two biological repeats; p values: * $p < 0.05$, ** $p < 0.01$, *** $p < 0.001$.

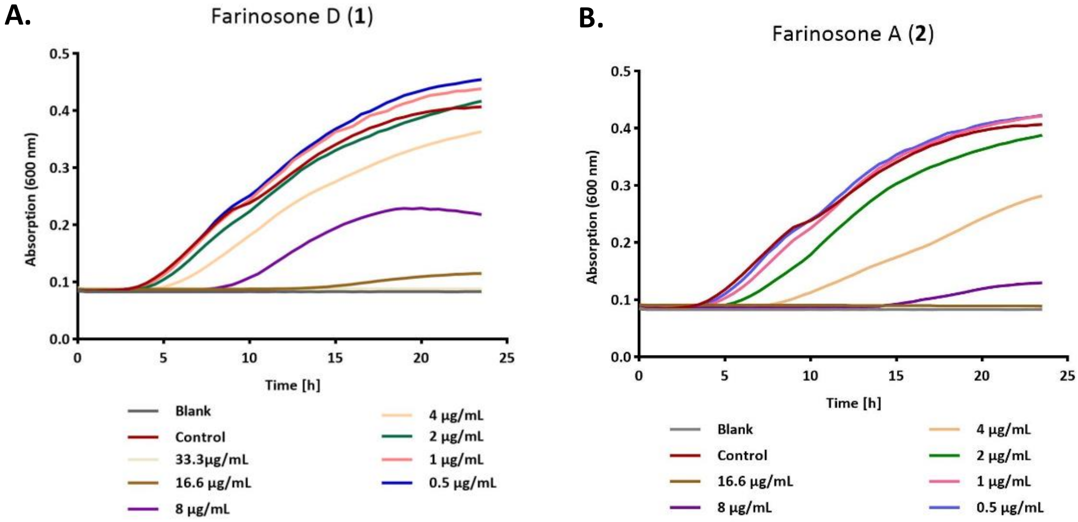

Figure S2. A. Effect of farinosone D (**1**) on growth of *S. aureus* at varying concentrations in Müller Hinton Broth (MHB) medium. B. Effect of farinosone A (**2**) on growth of *S. aureus* at varying concentrations in Müller Hinton Broth (MHB) medium.

Generic Display Report

Analysis Info

		Acquisition Date	18.06.2023 05:31:05
Analysis Name	S:\DATA\AmaZon\rto20_RitaToshe\23_06\MY07955-Rice-S1-F4-F03_BC5_01_14453.d		
Method	14453.m	Operator	lab
Sample Name	MY07955-Rice-S1-F4-F03	Instrument	amaZon speed
Comment			

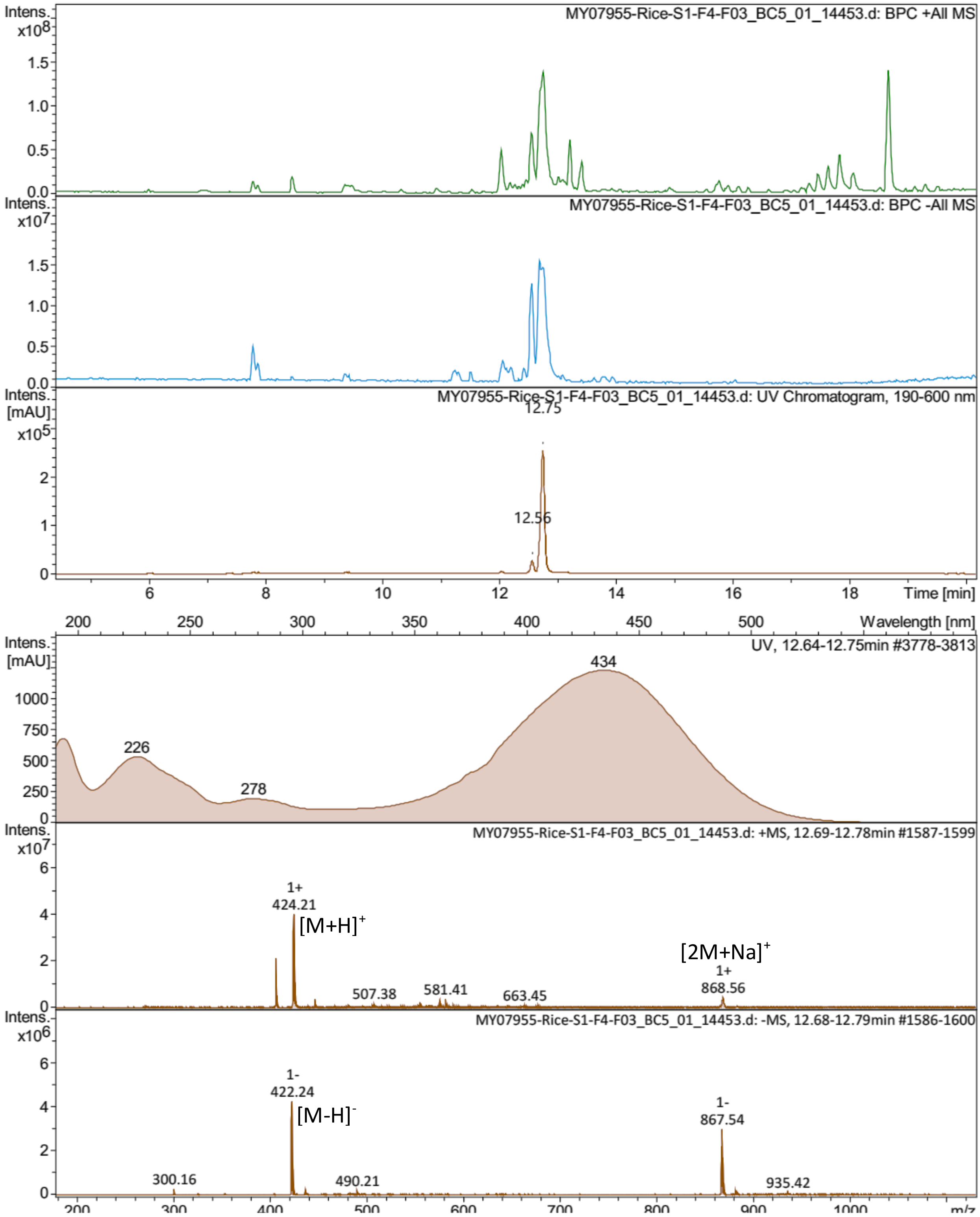

Bruker Compass DataAnalysis 4.4	printed: 21.06.2023 15:54:17	by: sel22	Page 1 of 1

Figure S3. LRESIMS spectrum of **1**.

Generic Display Report

Analysis Info

Analysis Name	S:\DATA\MaXis\rto20_Rita Toshe\23_06\MY07955-Rice-S1-F04-R2-F03_43_01_12047.d	Acquisition Date	28.06.2023 11:55:58
Method	pos_säure_10000_screening_ms_100_2500_line.m	Operator	ate06
Sample Name	MY07955-Rice-S1-F04-R2-F03	Instrument	maXis
Comment	Screening01<br>Waters Acquity UPLC BEH C_18 1,7um 2.1x50mm		

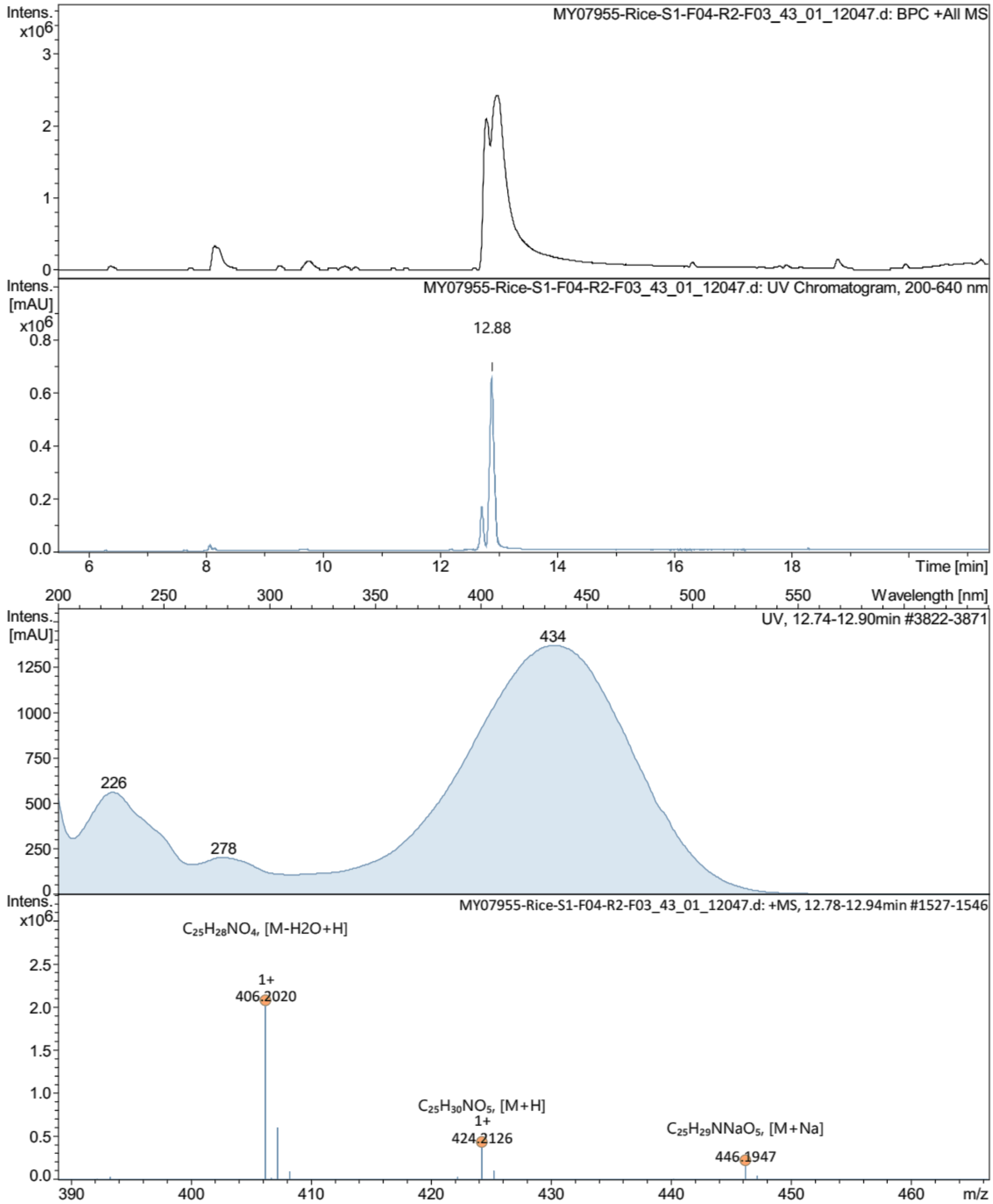

Bruker Compass DataAnalysis 4.4 printed: 14.07.2023 12:37:38 by: sel22 Page 1 of 1

Figure S4. HRESIMS spectrum of **1**.

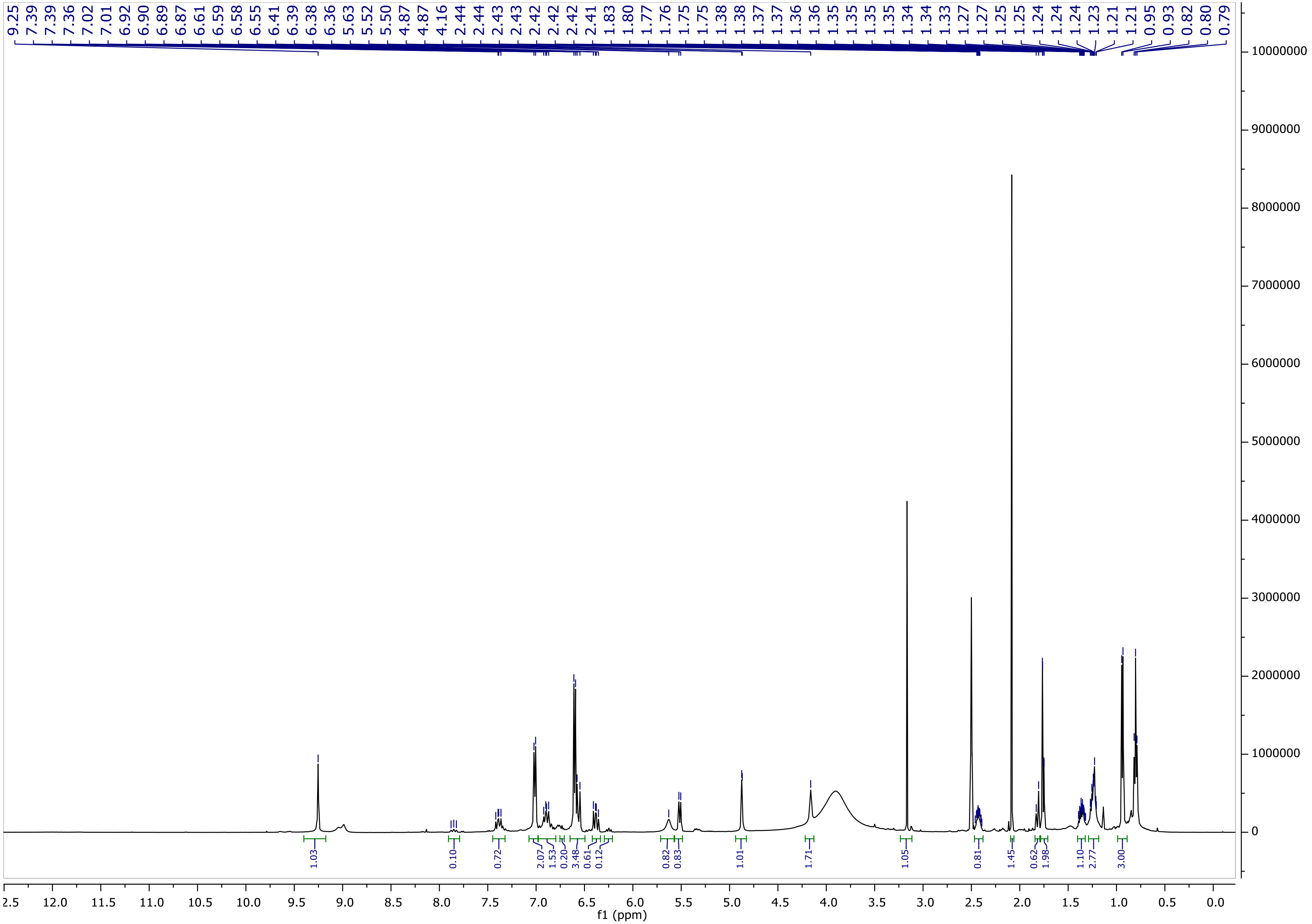

Figure S5. ^{1}H NMR spectrum of **1** in DMSO-d_6 at 500 MHz.

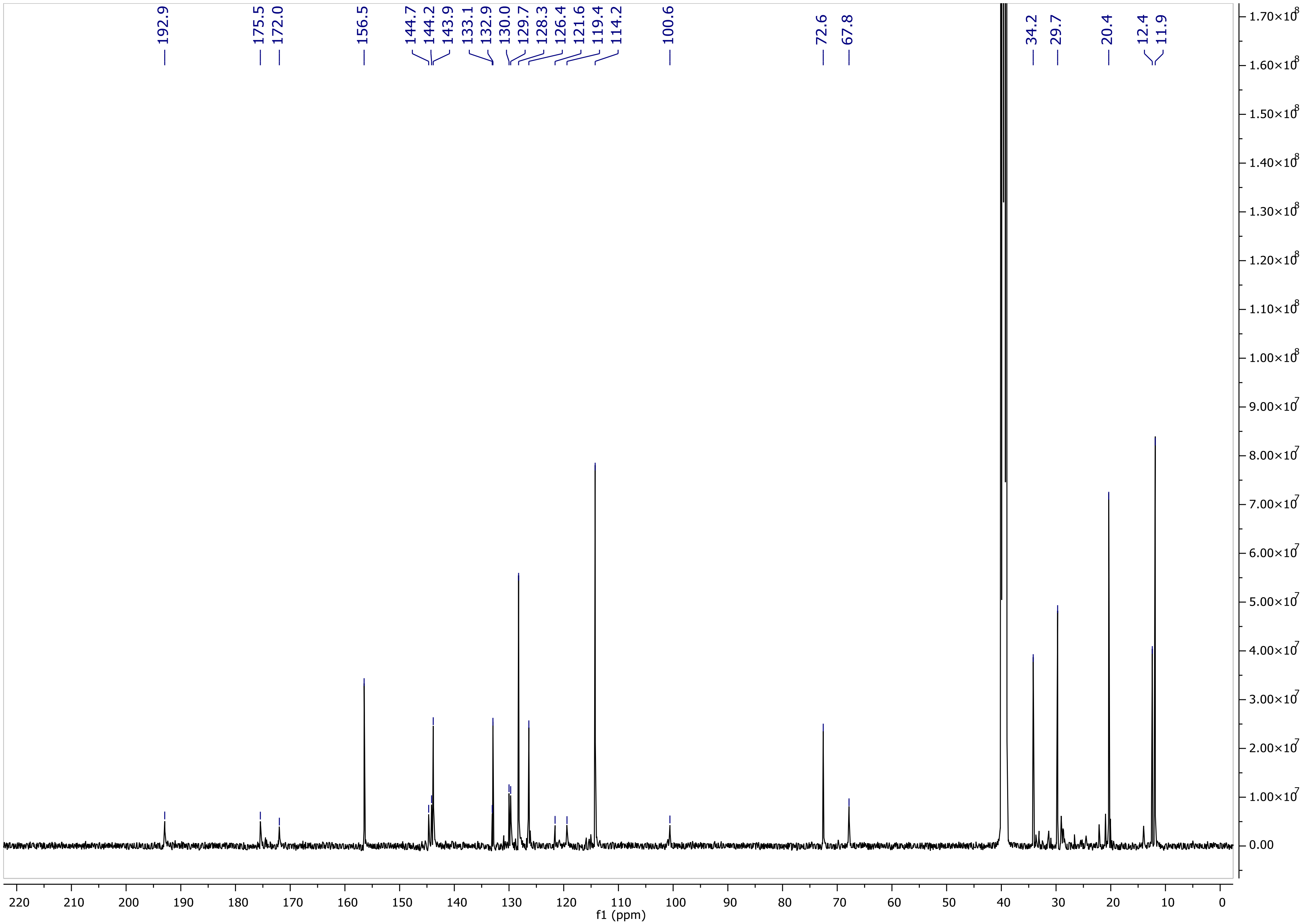

Figure S6. ^{13}C NMR spectrum of **1** in DMSO-d_6 at 125 MHz.

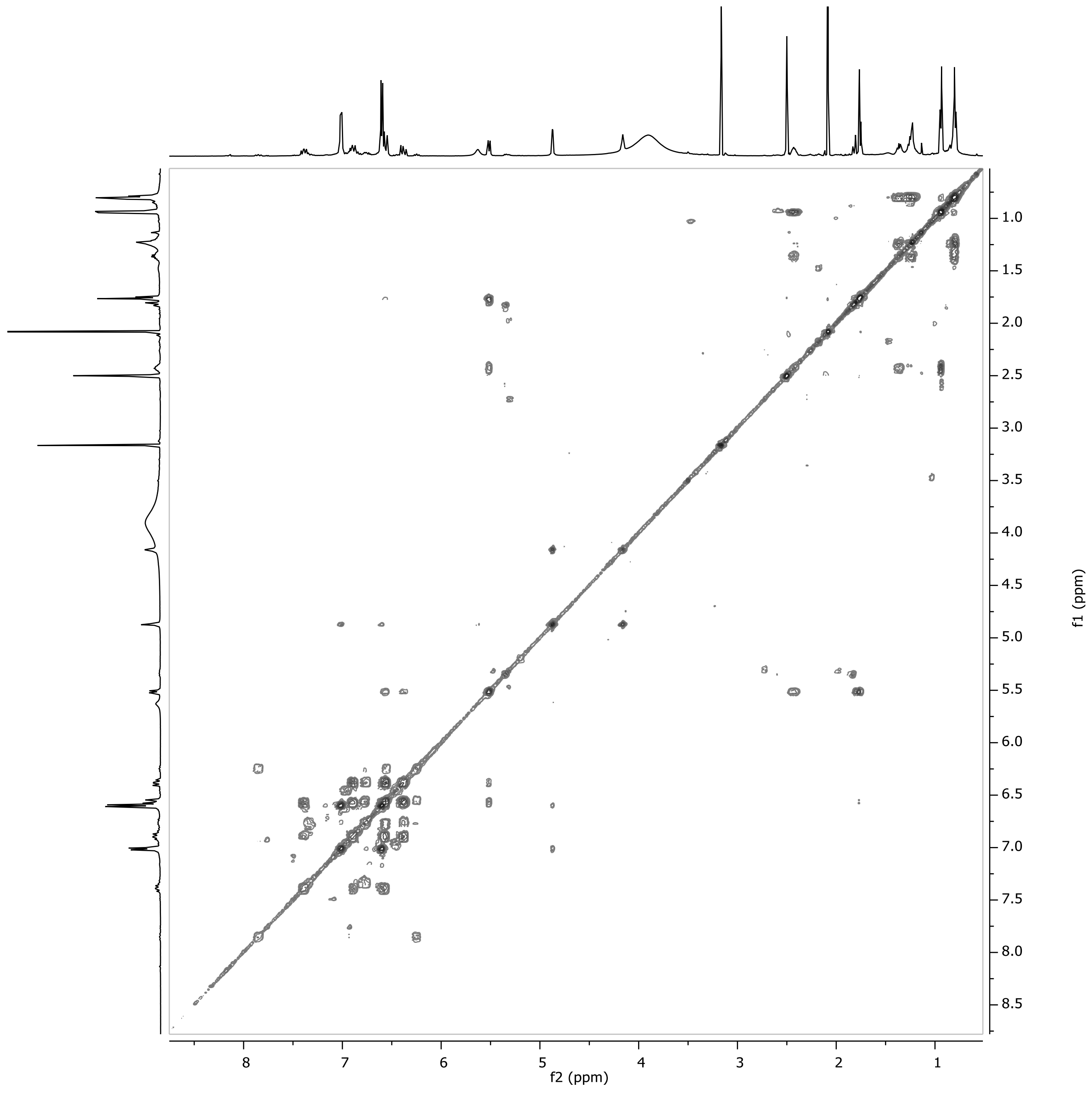

Figure S7. ^{1}H-^{1}H COSY spectrum of **1** in DMSO-d_6 at 500 MHz.

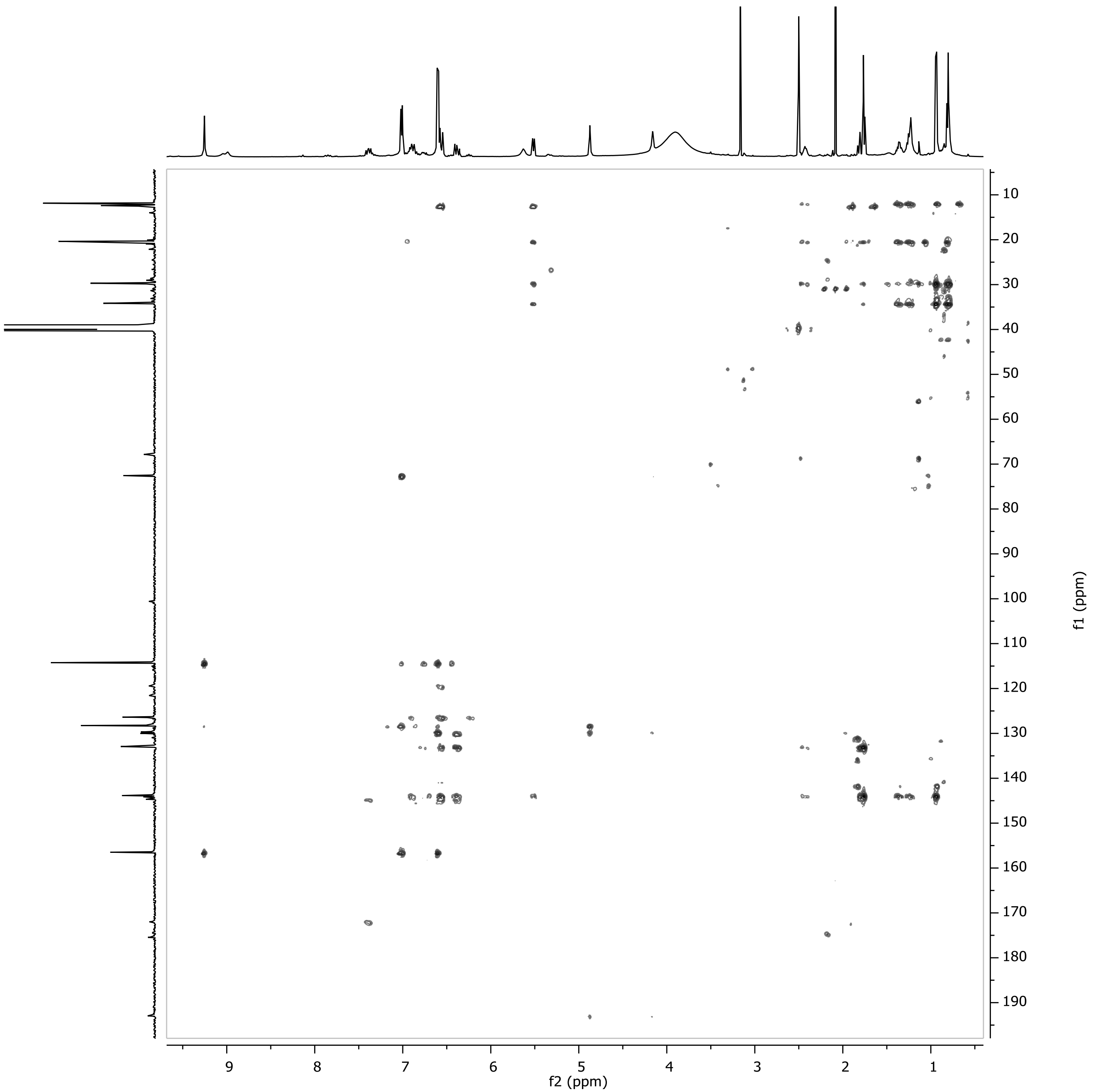

Figure S8. HMBC spectrum of **1** in DMSO-d_6 at 500 MHz.

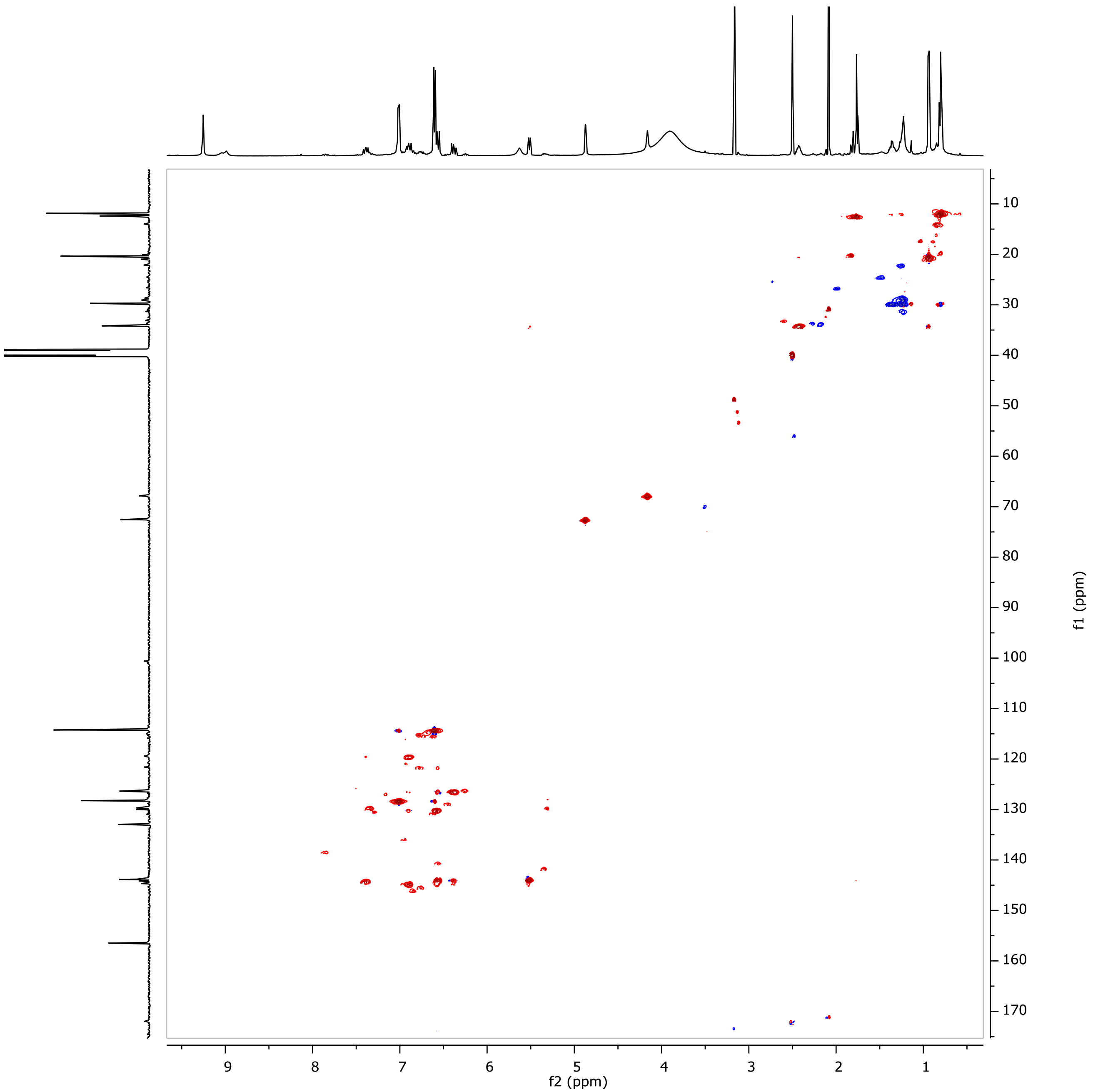

Figure S9. HSQC spectrum of **1** in DMSO-d_6 at 500 MHz.

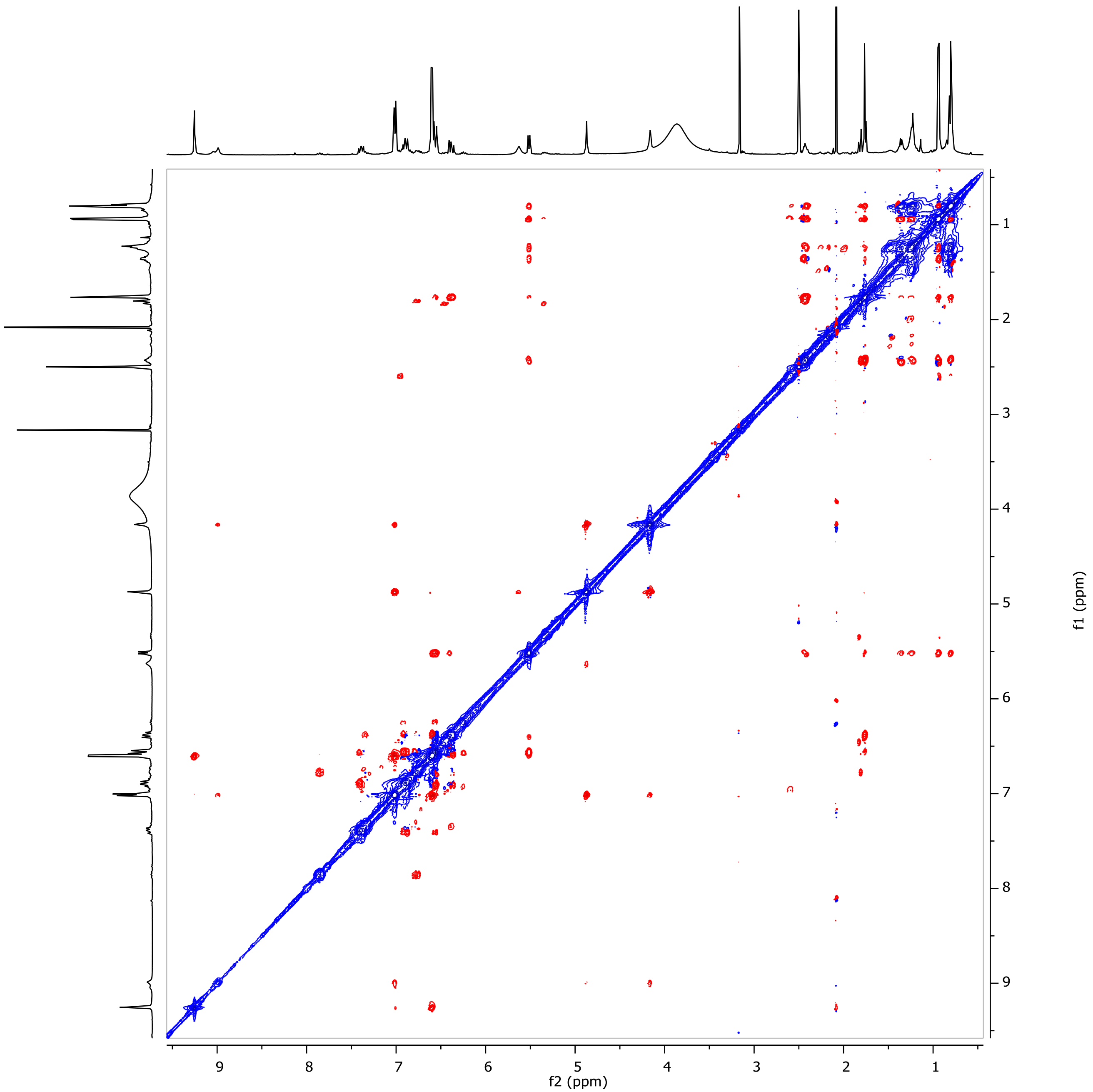

Figure S10. ROESY spectrum of **1** in DMSO-d_6 at 500 MHz.

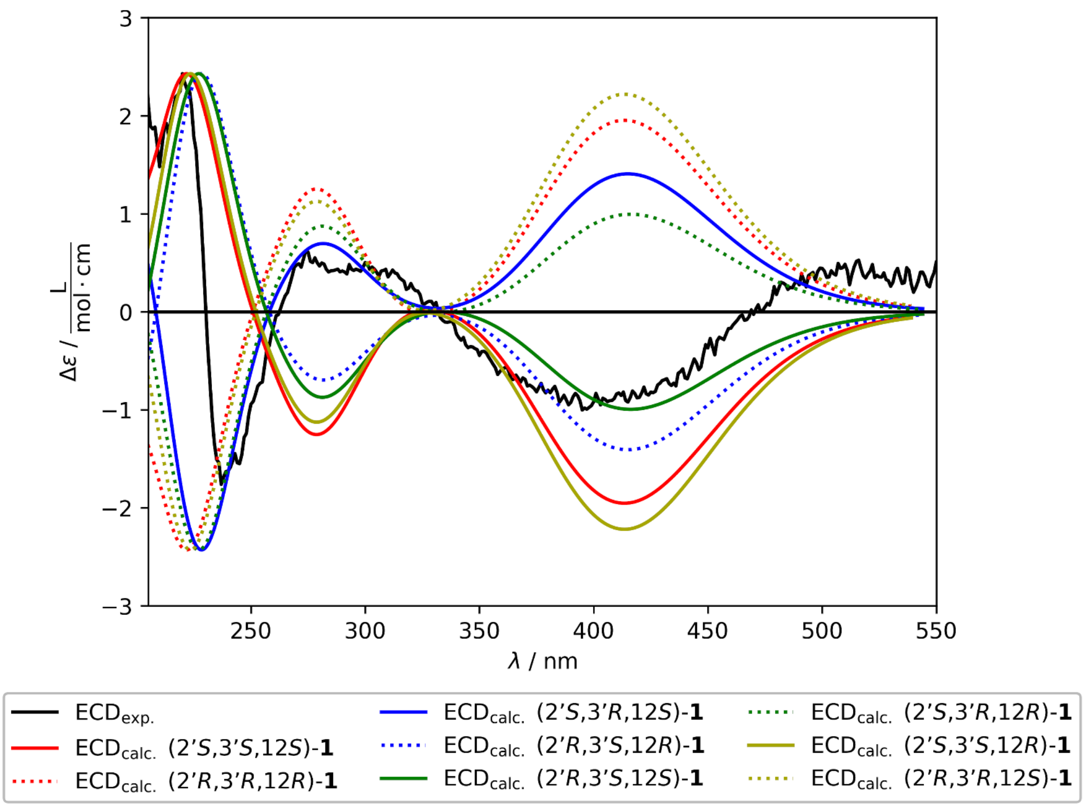

Figure S11. Comparison of experimental (black) and simulated Boltzmann-averaged ECD spectra for compound **1**.

Table S1. Similarity factor (Bruhn et al., 2013) for each calculated stereoisomer of compound **1**.

Stereoisomer of **1**	Similarity factor
(2'*S*,3'*S*,12*S*)	0.70
(2'*S*,3'*S*,12*R*)	0.69
(2'*R*,3'*S*,12*R*)	0.61
(2'*R*,3'*S*,12*S*)	0.60
(2'*S*,3'*R*,12*R*)	0.25
(2'*S*,3'*R*,12*S*)	0.24
(2'*R*,3'*R*,12*S*)	0.16
(2'*R*,3'*R*,12*R*)	0.16

Bruhn, T.; Schaumlöffel, A.; Hemberger, Y.; Bringmann, G. SpecDis: quantifying the comparison of calculated and experimental electronic circular dichroism spectra. *Chirality* **2013**, *25(4)*, 243–249. doi:10.1002/chir.22138

Generic Display Report

Analysis Info

Analysis Name	S:\DATA\MaXis\rto20_Rita Toshe\23_05\MY07955-Rice-S1-F6-R4-F1.2_32_01_11528.d	Acquisition Date	11.05.2023 02:56:01
Method	pos_säure_10000_screening_ms_100_2500_line.m	Operator	ate06
Sample Name	MY07955-Rice-S1-F6-R4-F1.2	Instrument	maXis
Comment	Screening01<br>Waters Acquity UPLC BEH C_18 1,7um 2.1x50mm		

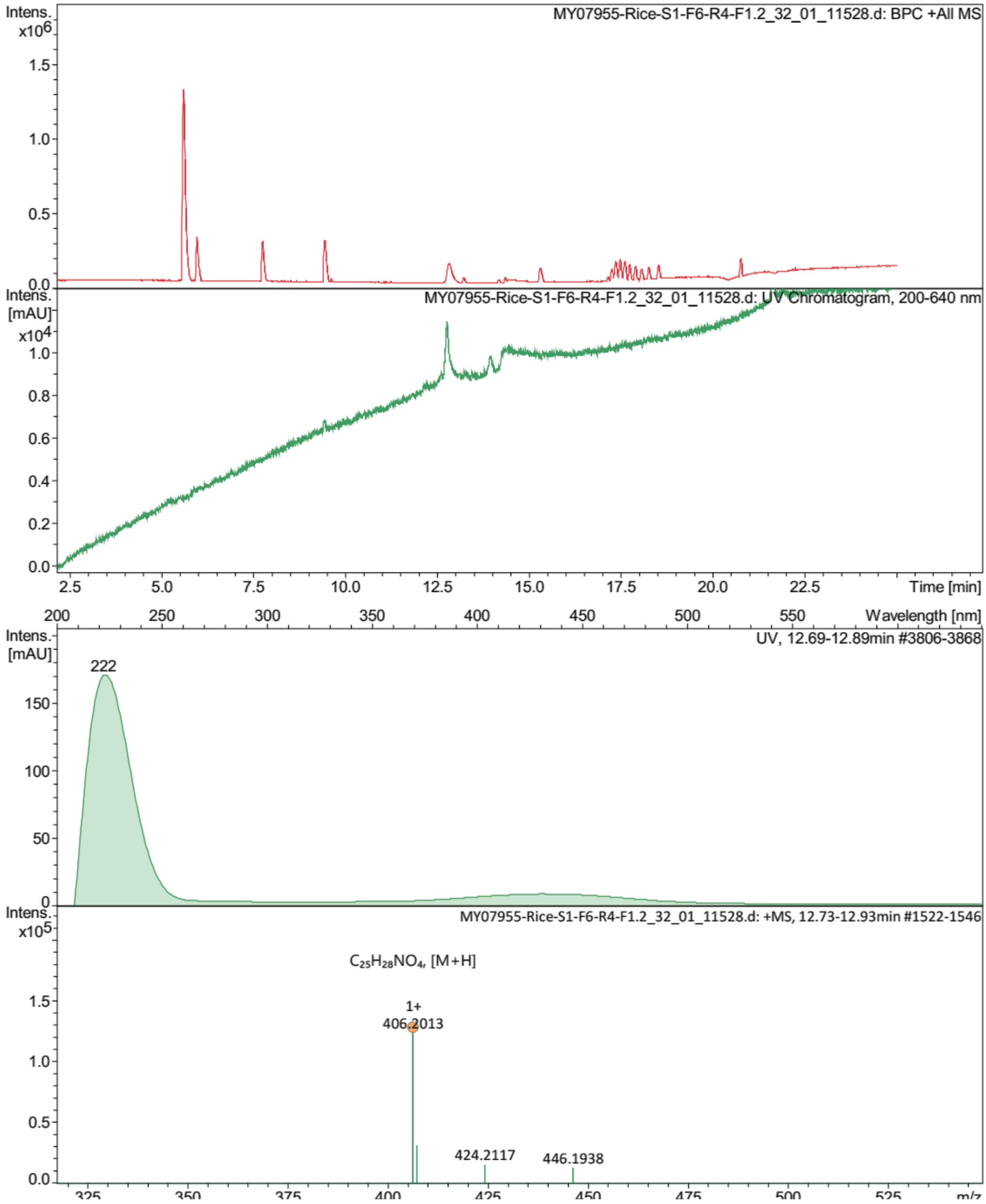

Bruker Compass DataAnalysis 4.4 printed: 13.05.2023 08:40:48 by: sel22 Page 1 of 1

Figure S12. HRESIMS spectrum of **2**.

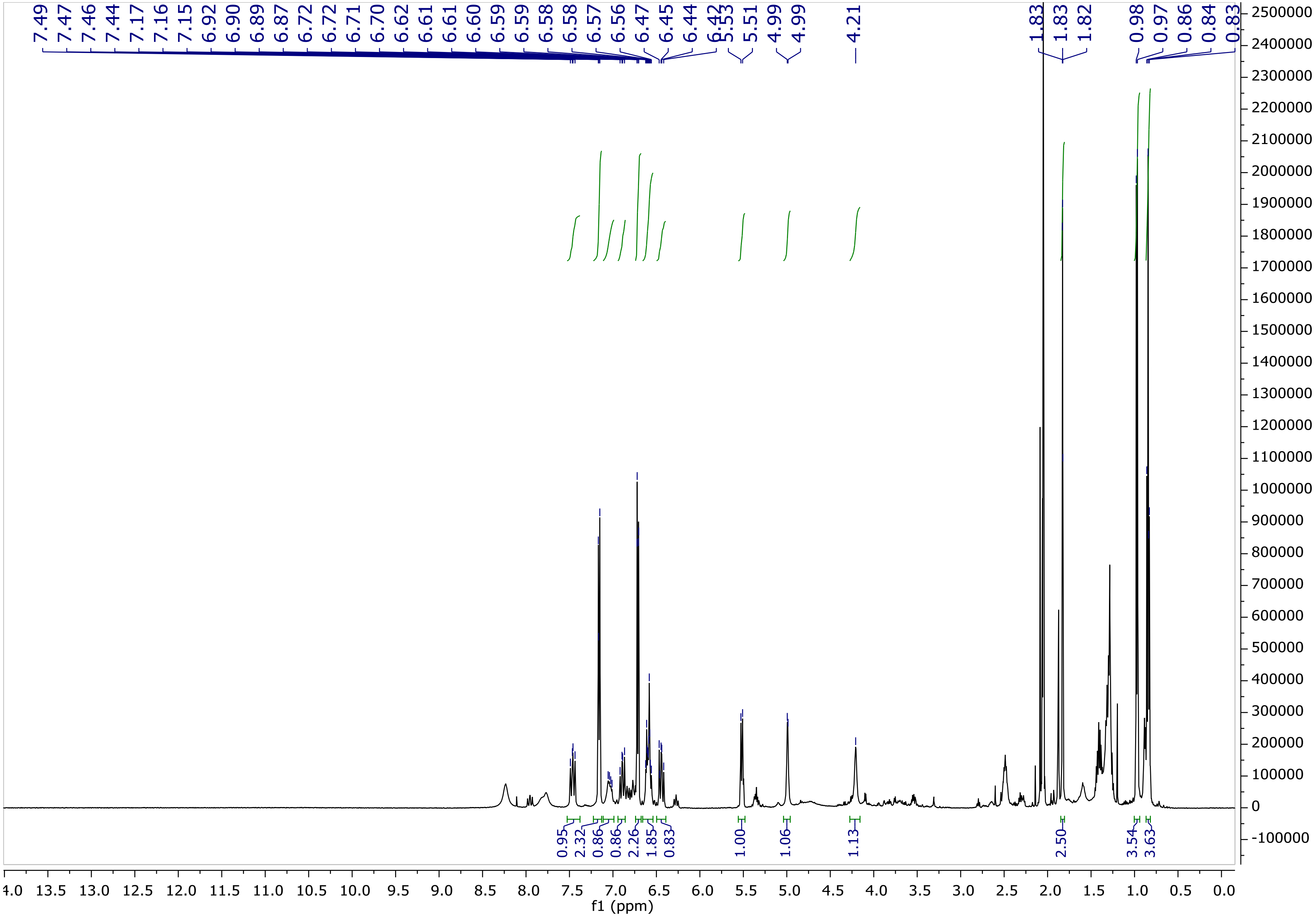

Figure S13. ^{1}H NMR spectrum of **2** in acetone-d_6 at 500 MHz.

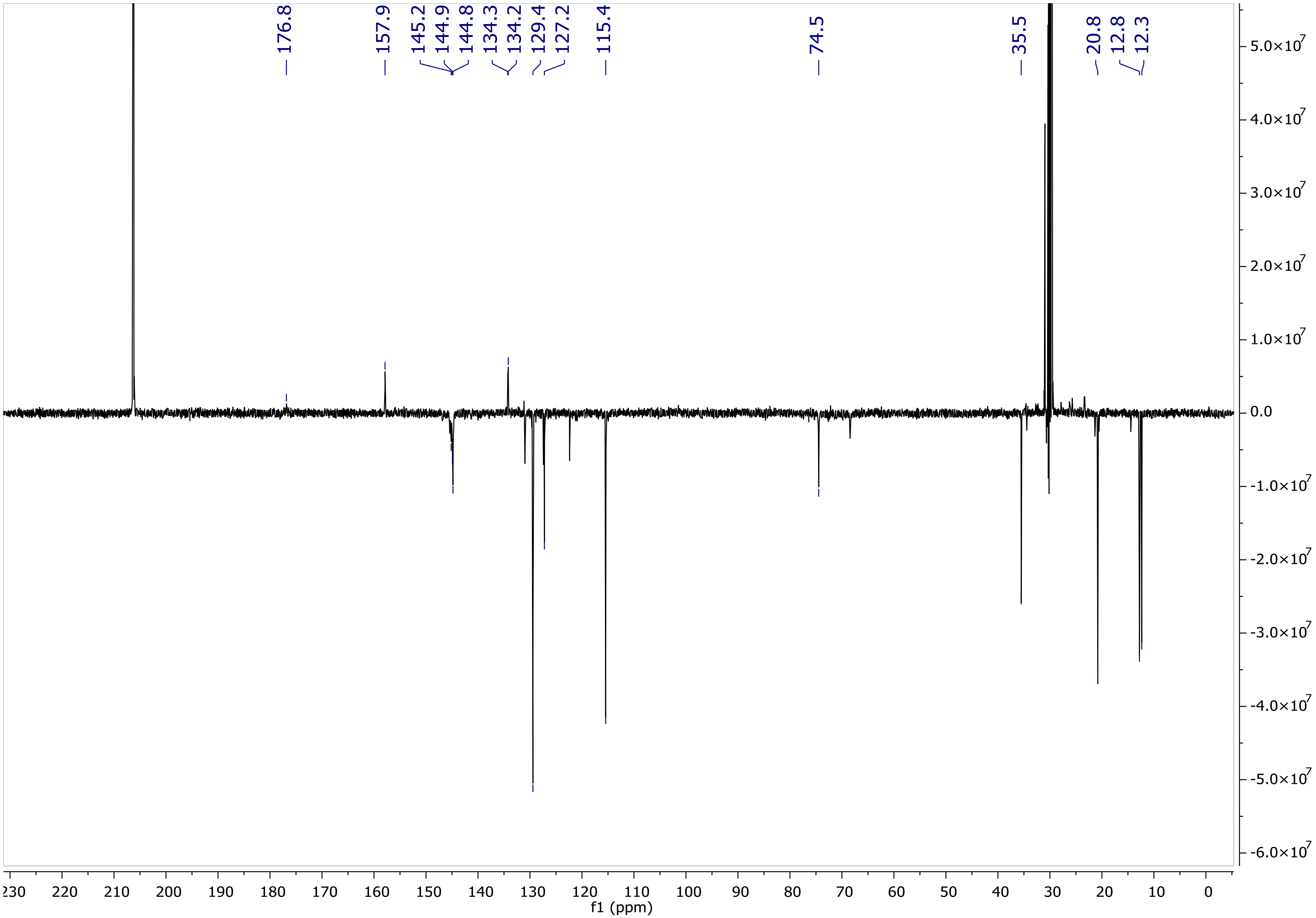

Figure S14. DEPTQ spectrum of **2** in acetone-d_6 at 125 MHz.

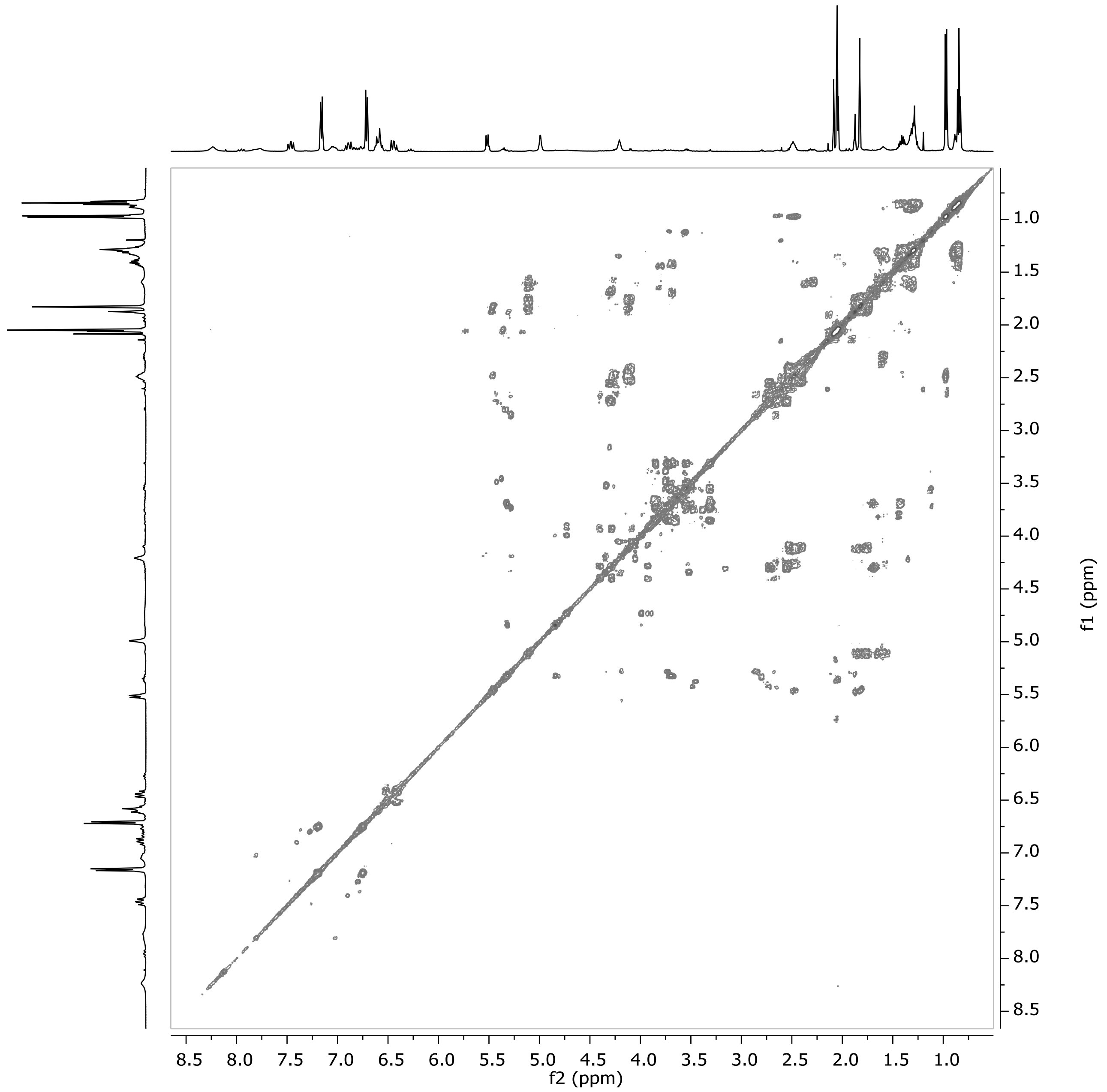

Figure S15. ^{1}H-^{1}H COSY spectrum of **2** in acetone-d_6 at 500 MHz.

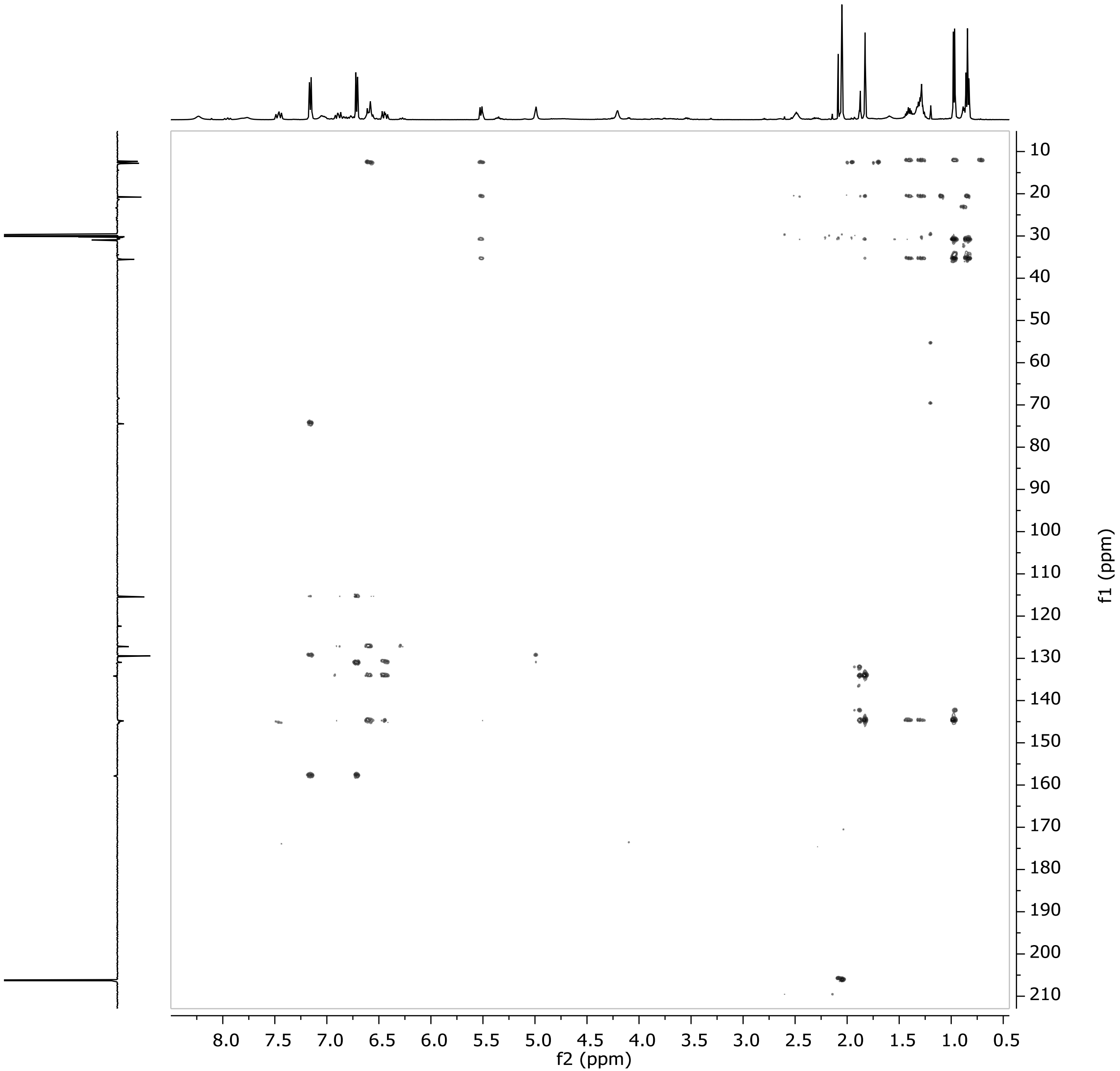

Figure S16. HMBC spectrum of **2** in acetone-d_6 at 500 MHz.

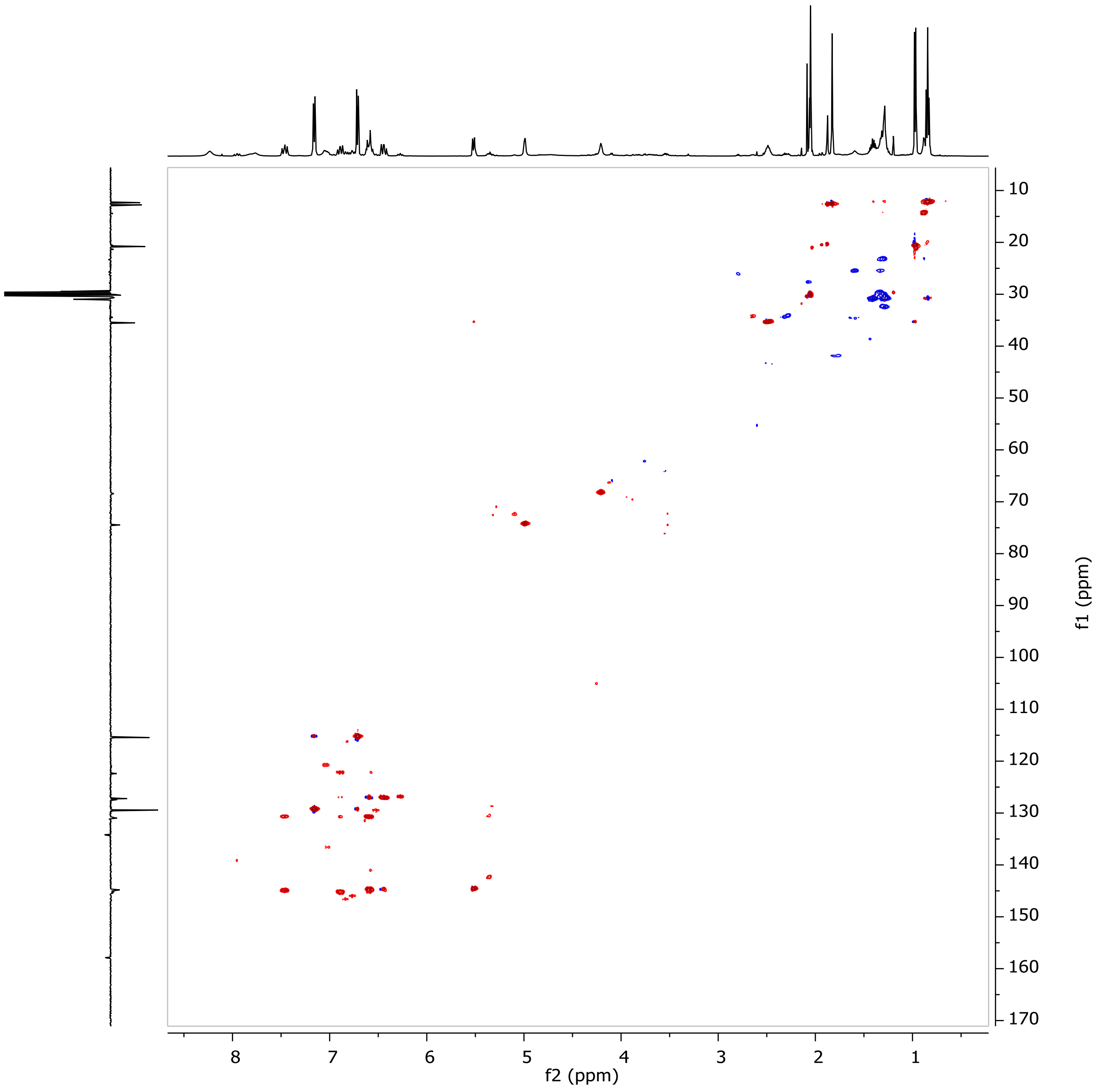

Figure S17. HSQC spectrum of **2** in acetone-d_6 at 500 MHz.

Generic Display Report

Analysis Info		Acquisition Date	11.05.2023 03:58:02
Analysis Name	S:\DATA\MaXis\rto20_Rita Toshe\23_05\MY07955-Rice-S1-F6-R4-F2.2_34_01_11530.d		
Method	pos_säure_10000_screening_ms_100_2500_line.m	Operator	ate06
Sample Name	MY07955-Rice-S1-F6-R4-F2.2	Instrument	maXis
Comment	Screening01<br>Waters Acquity UPLC BEH C_18 1,7um 2.1x50mm		

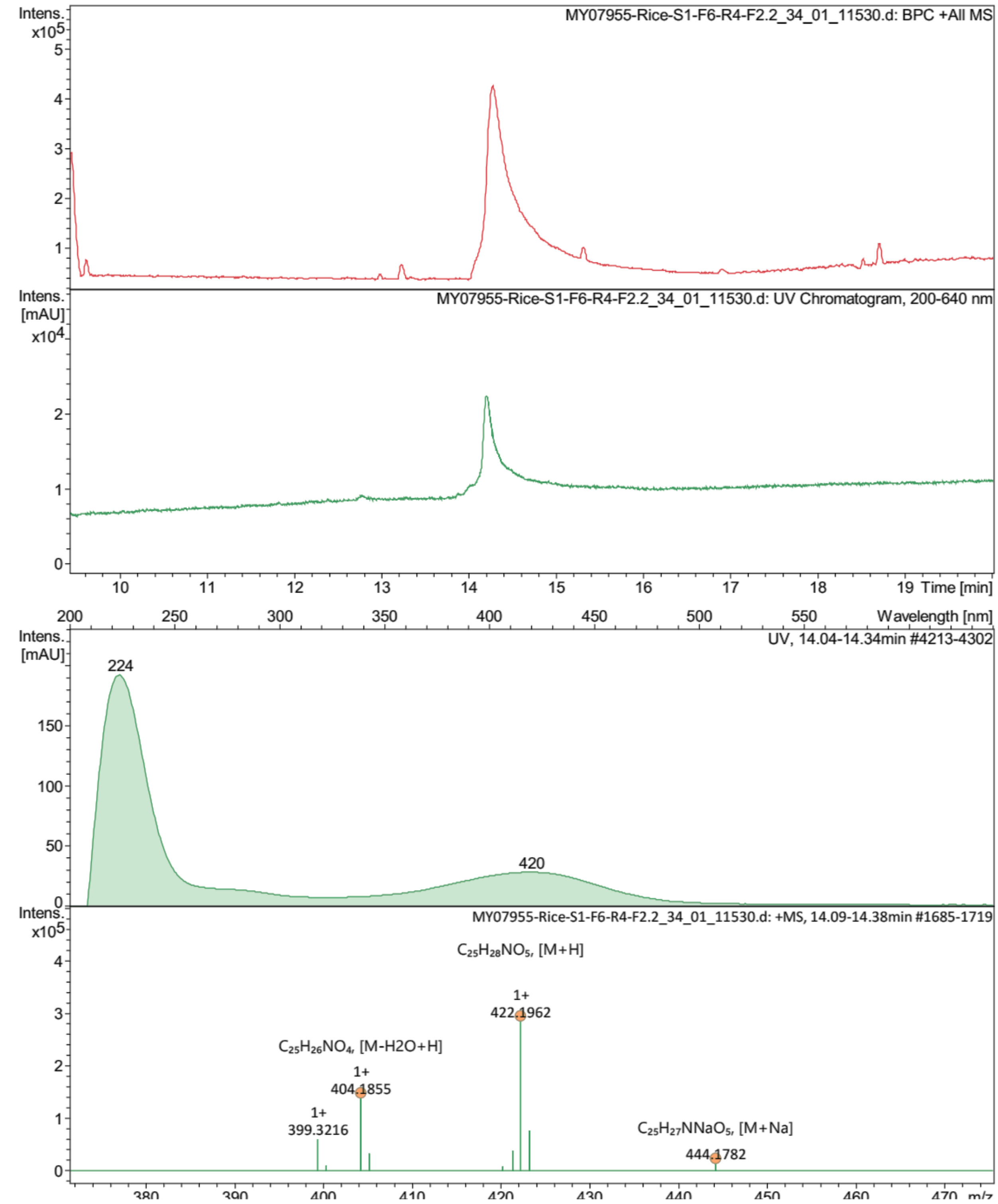

Bruker Compass DataAnalysis 4.4 printed: 13.05.2023 08:23:01 by: sel22 Page 1 of 1

Figure S18. HRESIMS spectrum of **3**.

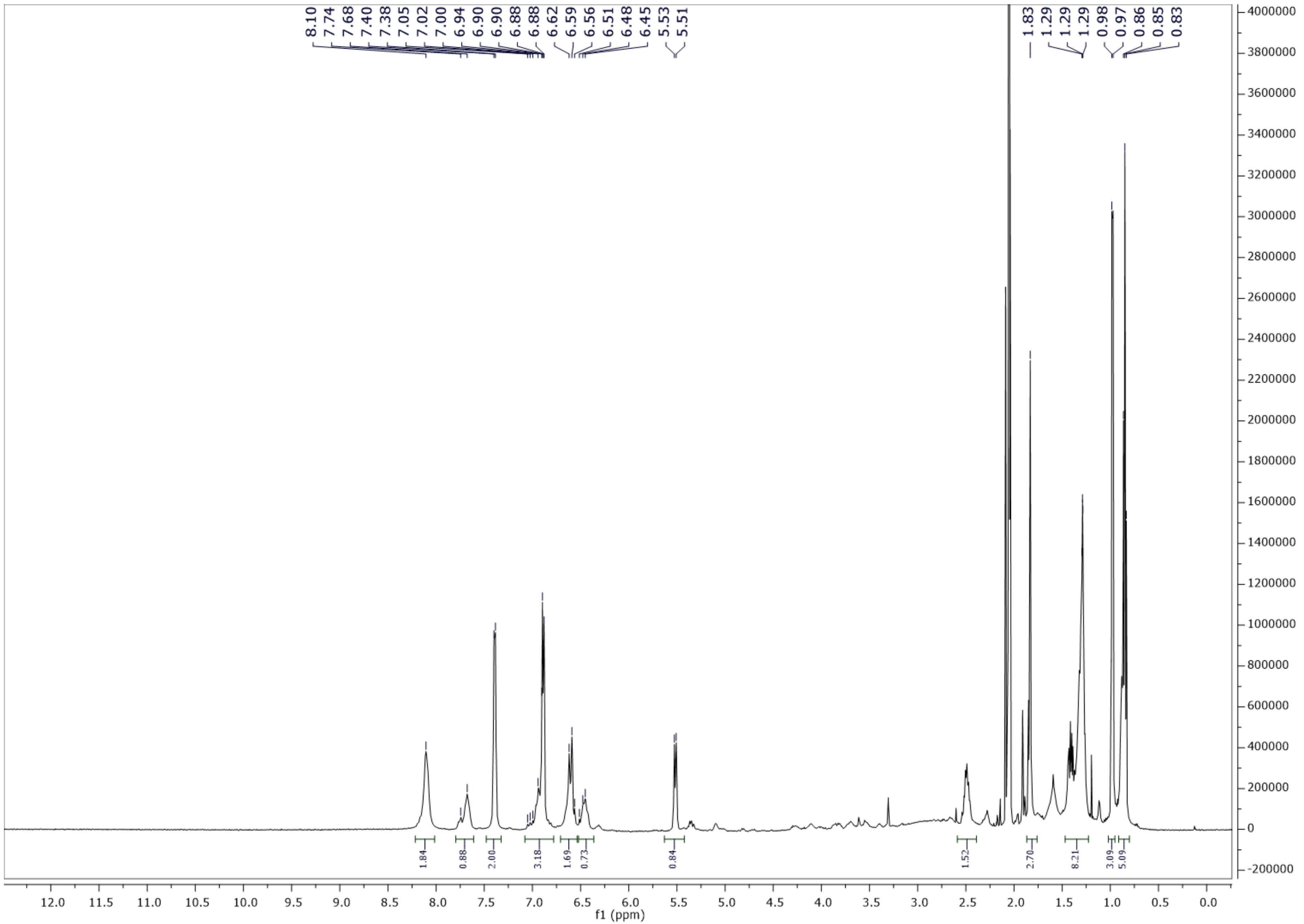

Figure S19. ^{1}H NMR spectrum of **3** in acetone-d_6 at 500 MHz.

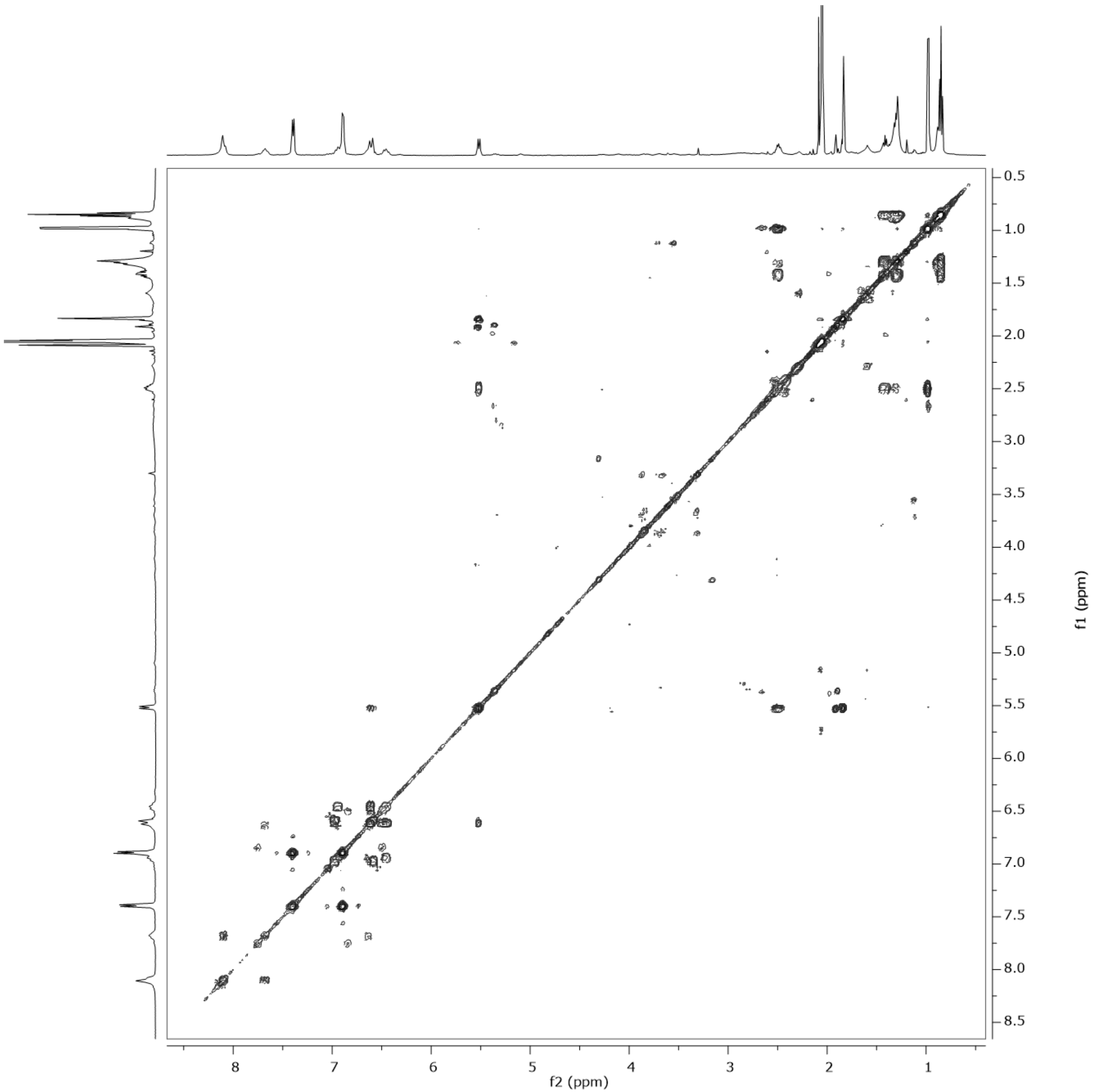

Figure S20. ^1H-^1H COSY spectrum of **3** in acetone-d_6 at 500 MHz.

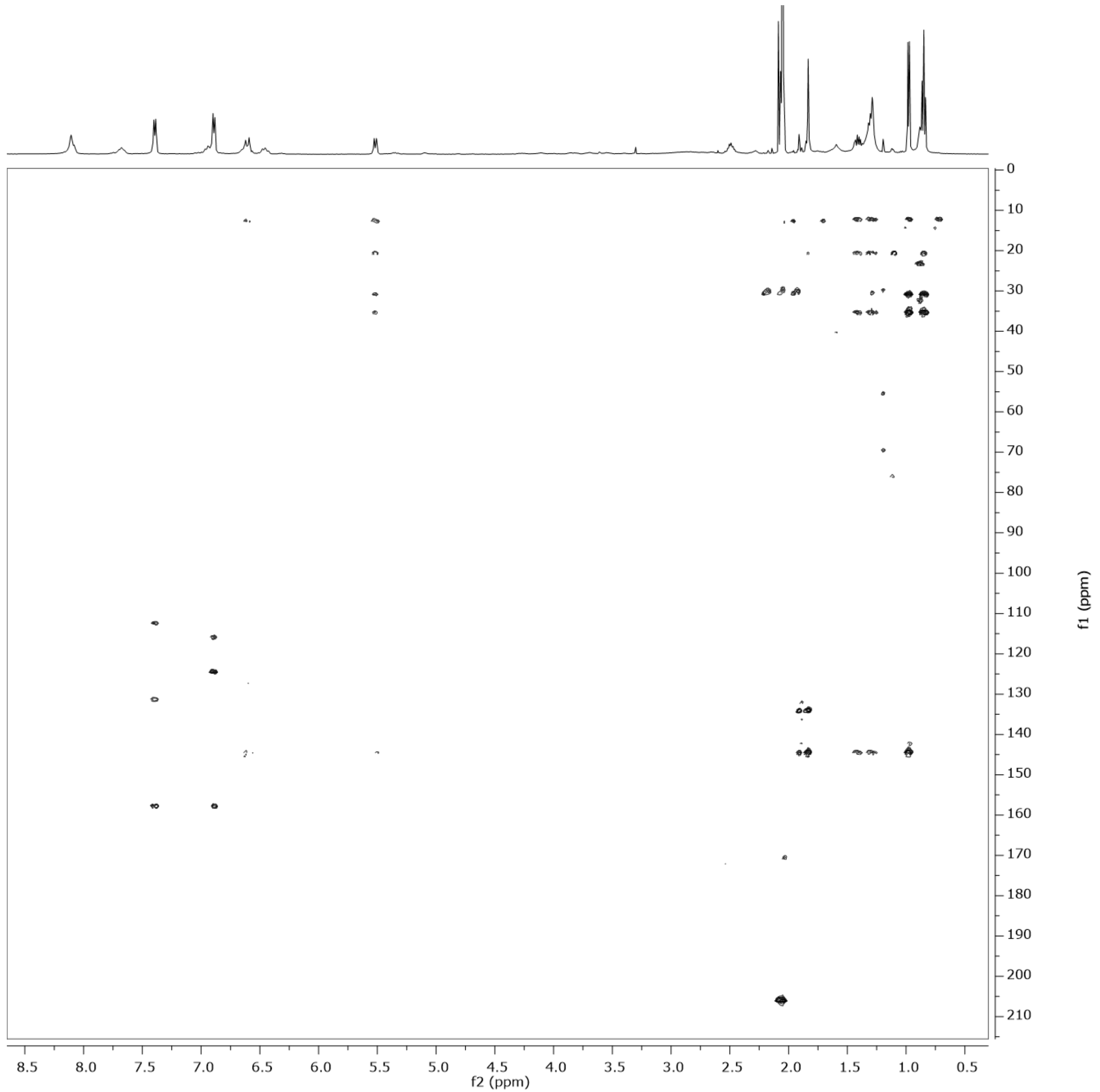

Figure S21. HMBC spectrum of **3** in acetone-d_6 at 500 MHz.

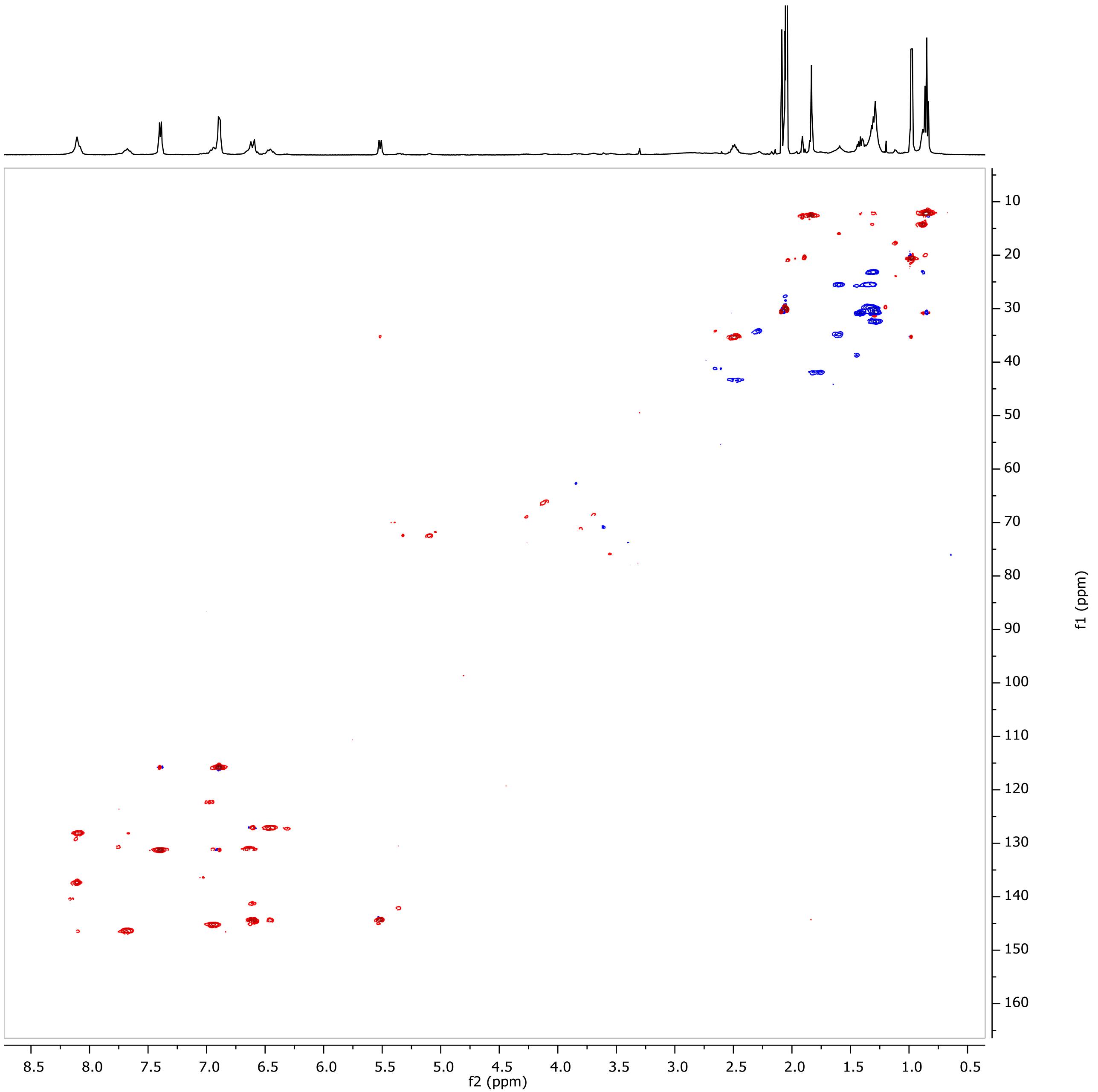

Figure S22. HSQC spectrum of **3** in acetone-d_6 at 500 MHz.

Generic Display Report

Analysis Info

Analysis Name	S:\DATA\MaXis\sel22_SherifElsayed\23_07\F08-F08_5_01_12318.d	Acquisition Date	12.07.2023 17:10:50
Method	pos_säure_10000_screening_ms_100_2500_line.m	Operator	ate06
Sample Name	F08-F08	Instrument	maXis
Comment	Screening01<br>Waters Acquity UPLC BEH C_18 1,7um 2.1x50mm		

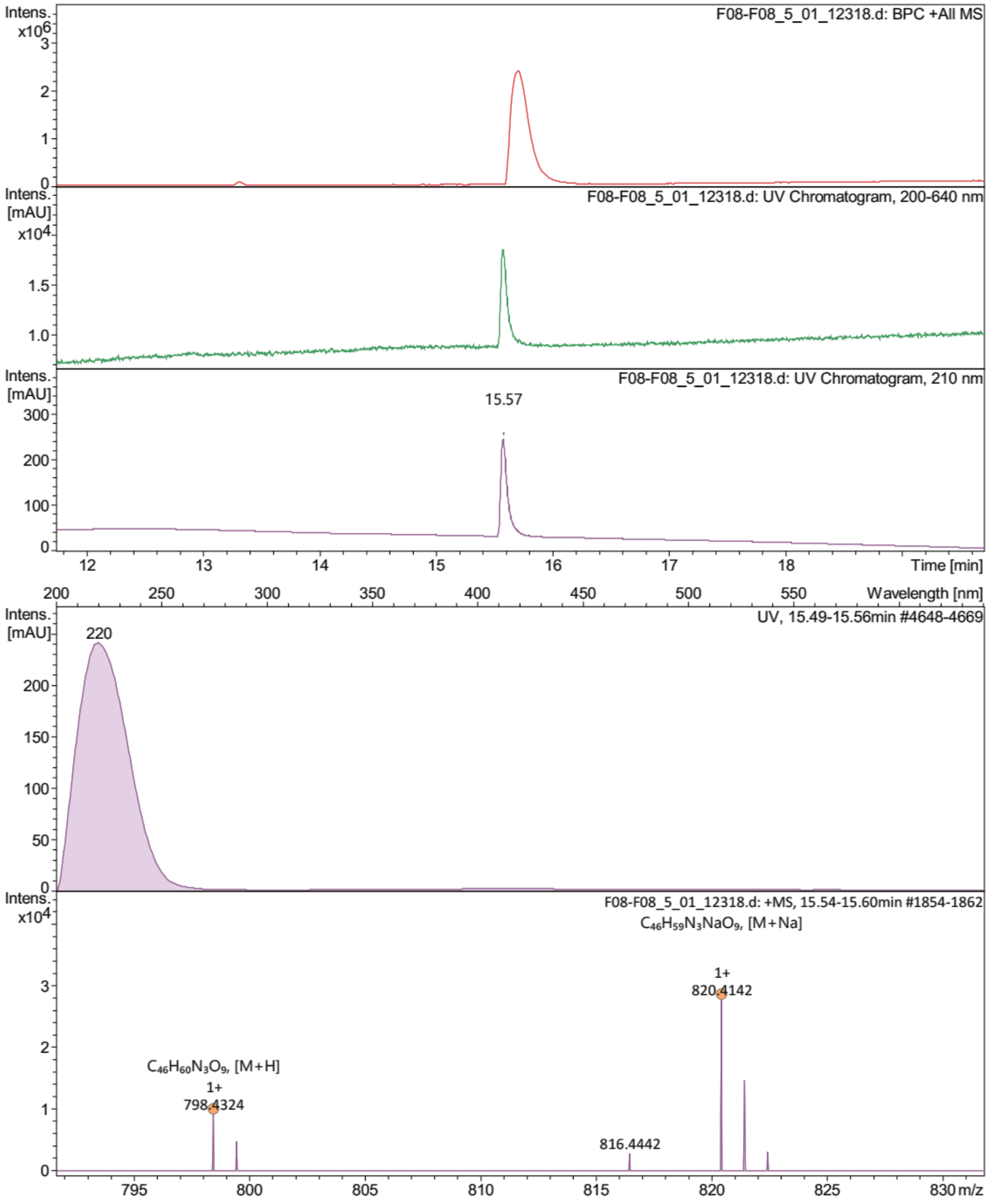

Bruker Compass DataAnalysis 4.4 printed: 14.07.2023 10:17:34 by: sel22 Page 1 of 1

Figure S23. HRESIMS spectrum of **4**.

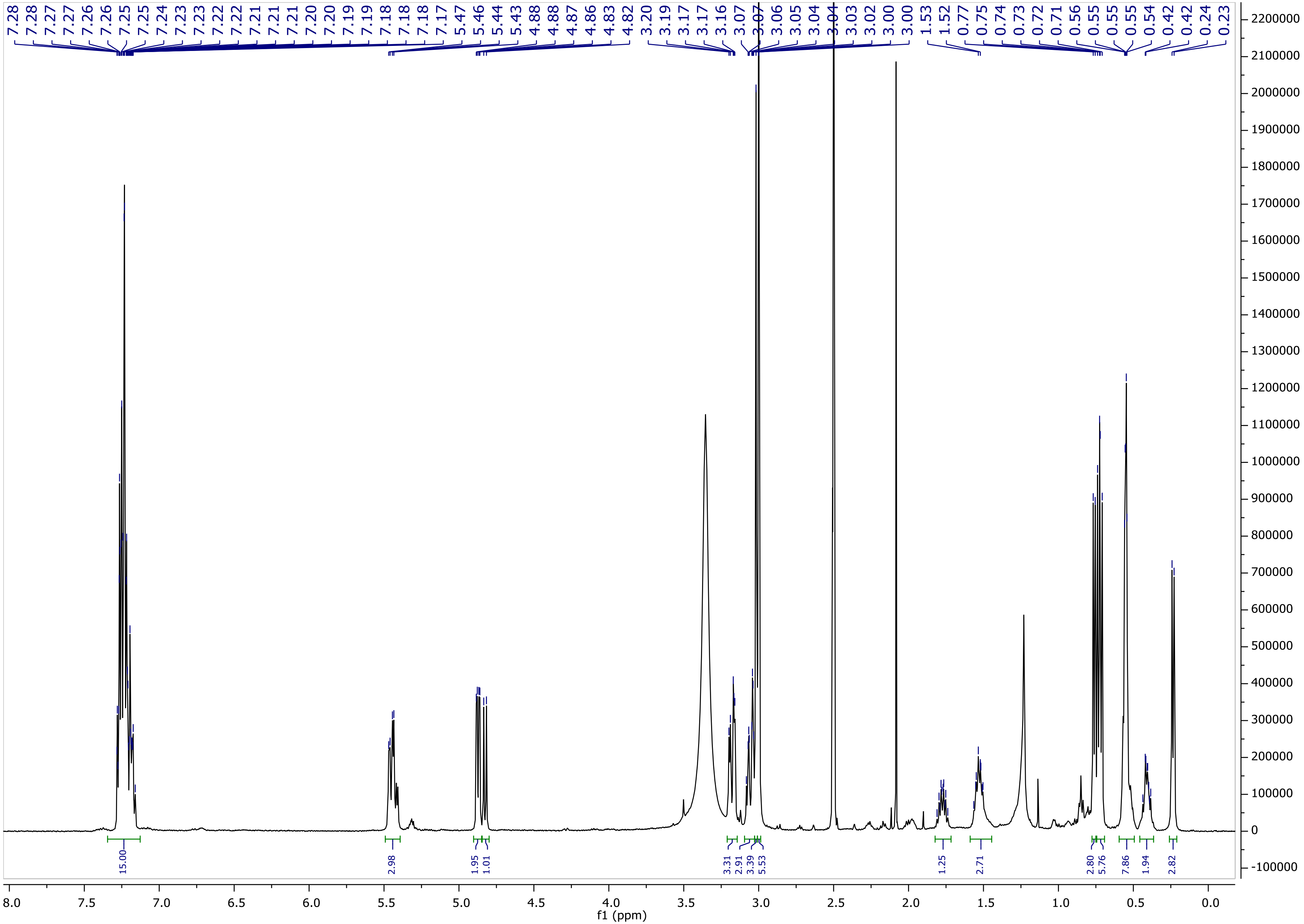

Figure S24. ^{1}H NMR spectrum of **4** in DMSO-d_6 at 500 MHz.

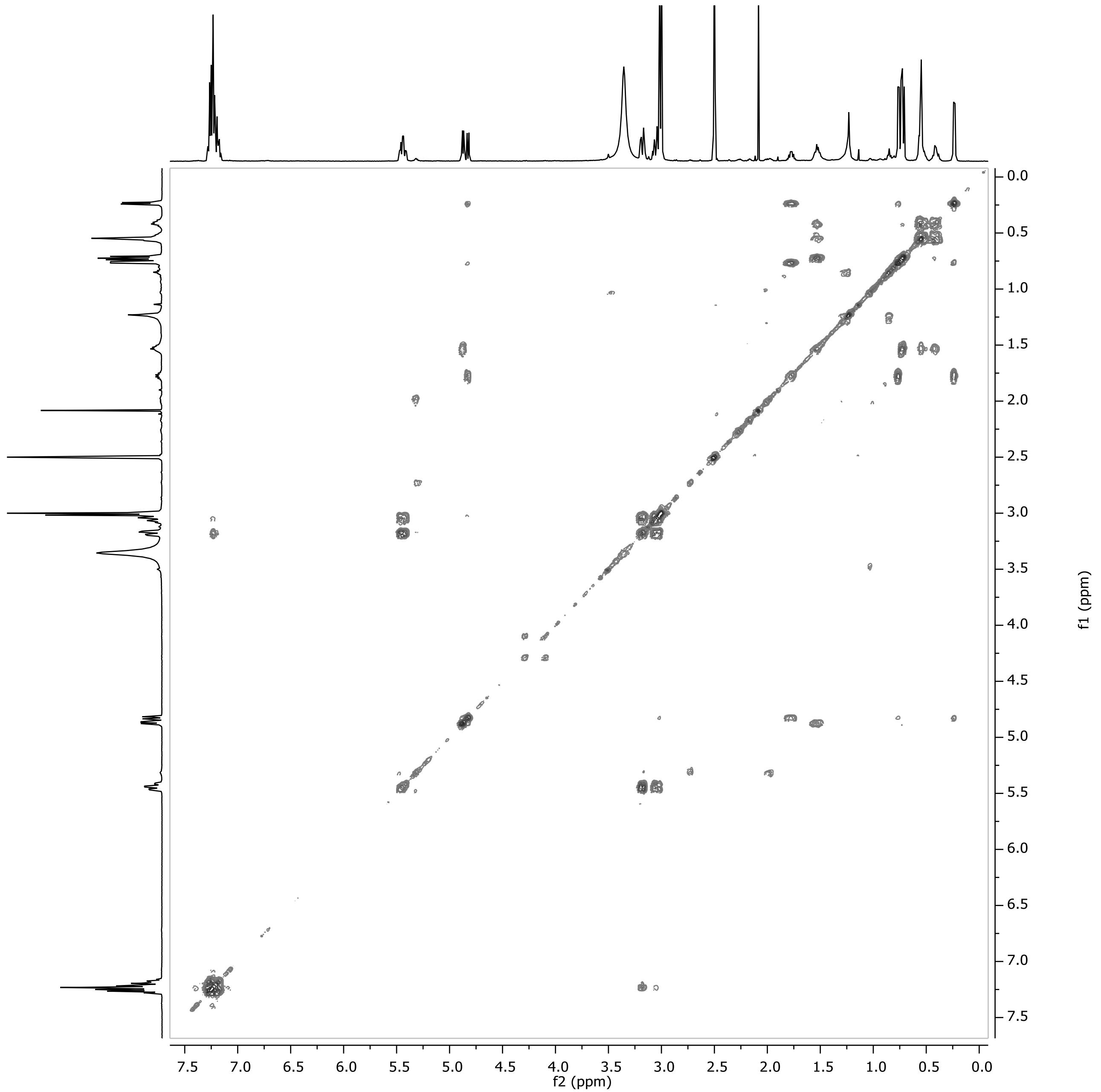

Figure S25. ^{1}H-^{1}H COSY spectrum of **4** in DMSO-d_6 at 500 MHz.

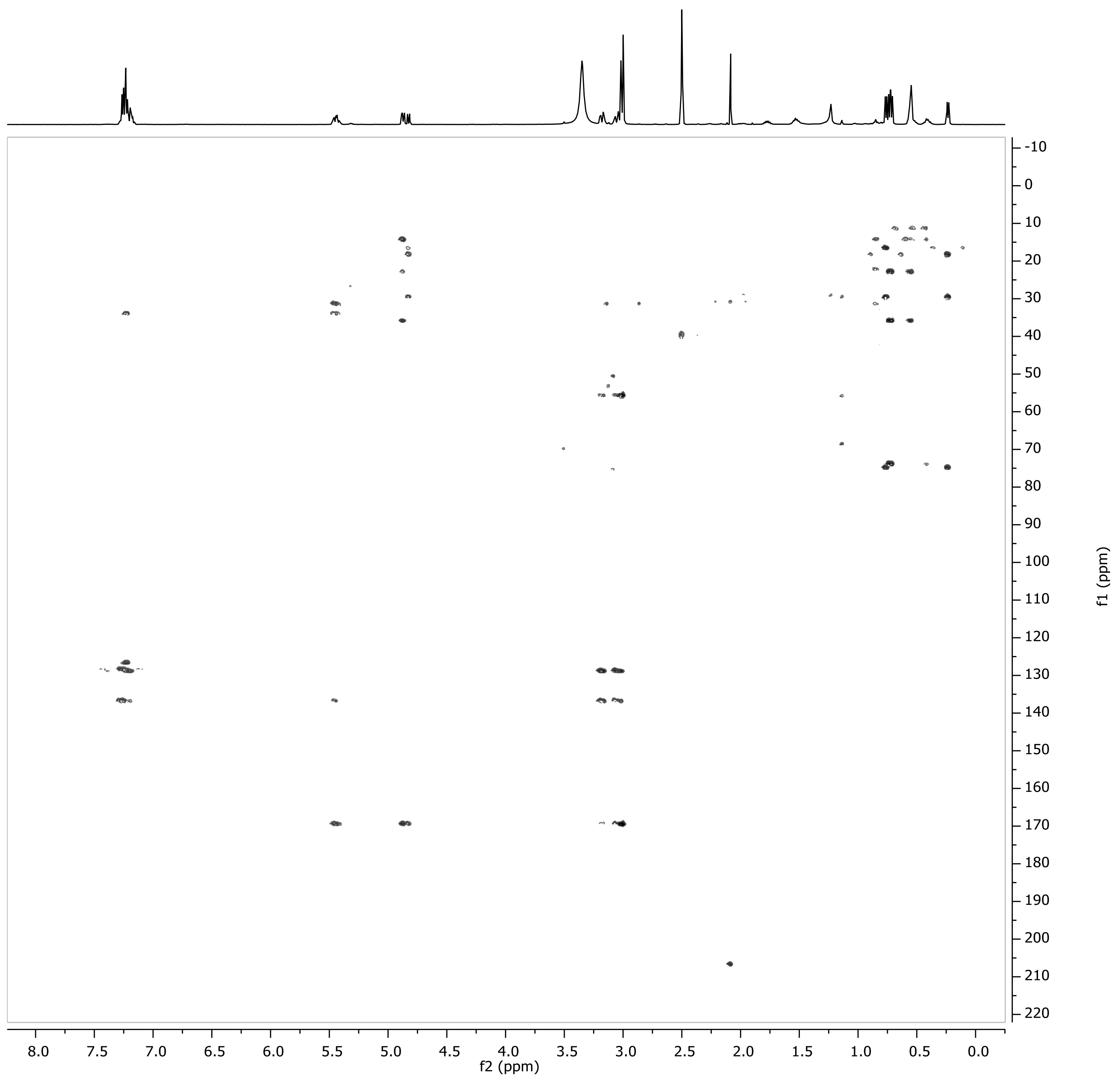

Figure S26. HMBC spectrum of **4** in DMSO-d_6 at 500 MHz.

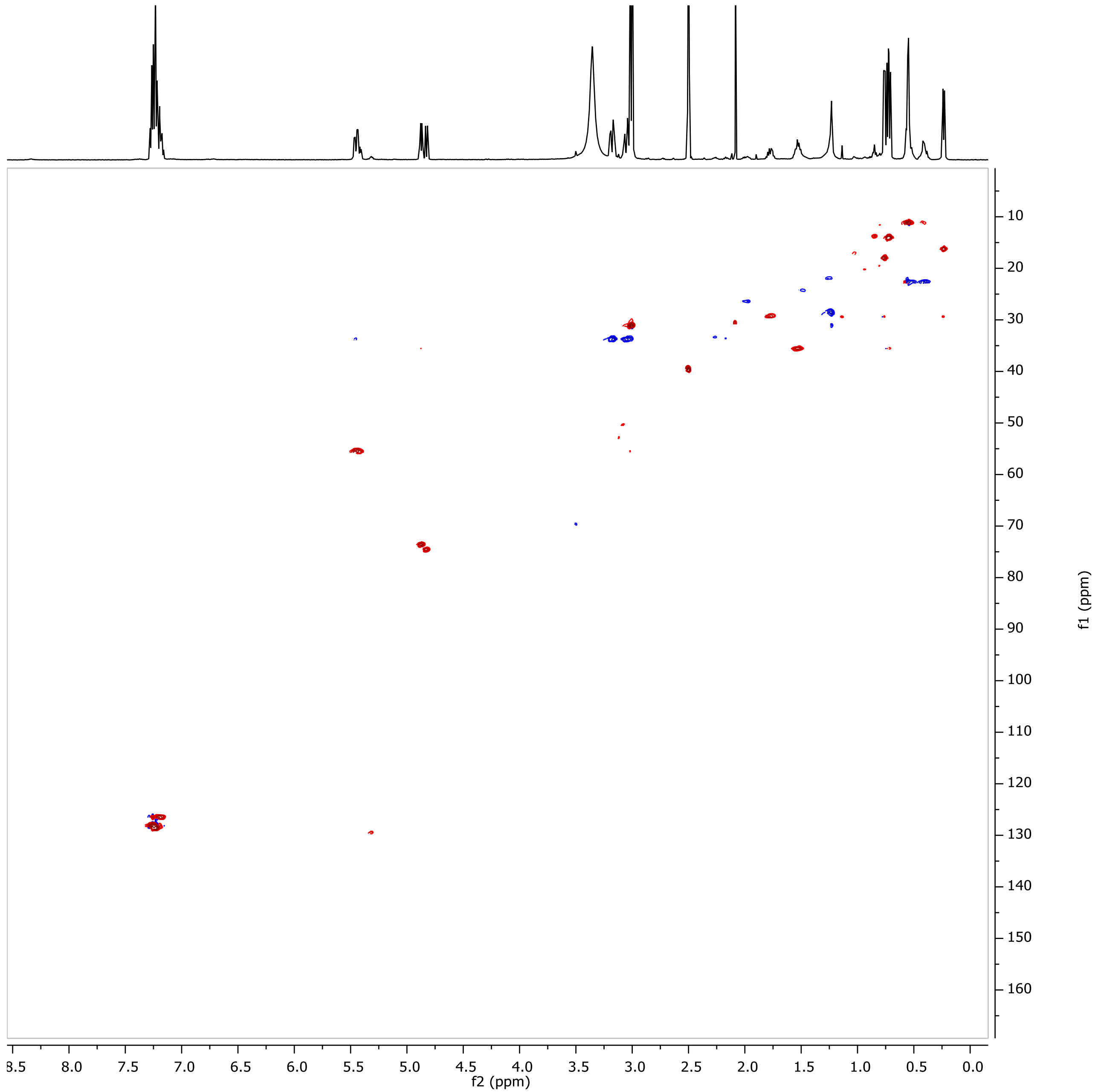

Figure S27. HSQC spectrum of **4** in DMSO-d_6 at 500 MHz.

Generic Display Report

**Analysis Info**		Acquisition Date	12.07.2023 16:39:53
Analysis Name	S:\DATA\MaXis\sel22_SherifElsayed\23_07\F04-F13_4_01_12317.d		
Method	pos_säure_10000_screening_ms_100_2500_line.m	Operator	ate06
Sample Name	F04-F13	Instrument	maXis
Comment	Screening01<br>Waters Acquity UPLC BEH C_18 1,7um 2.1x50mm		

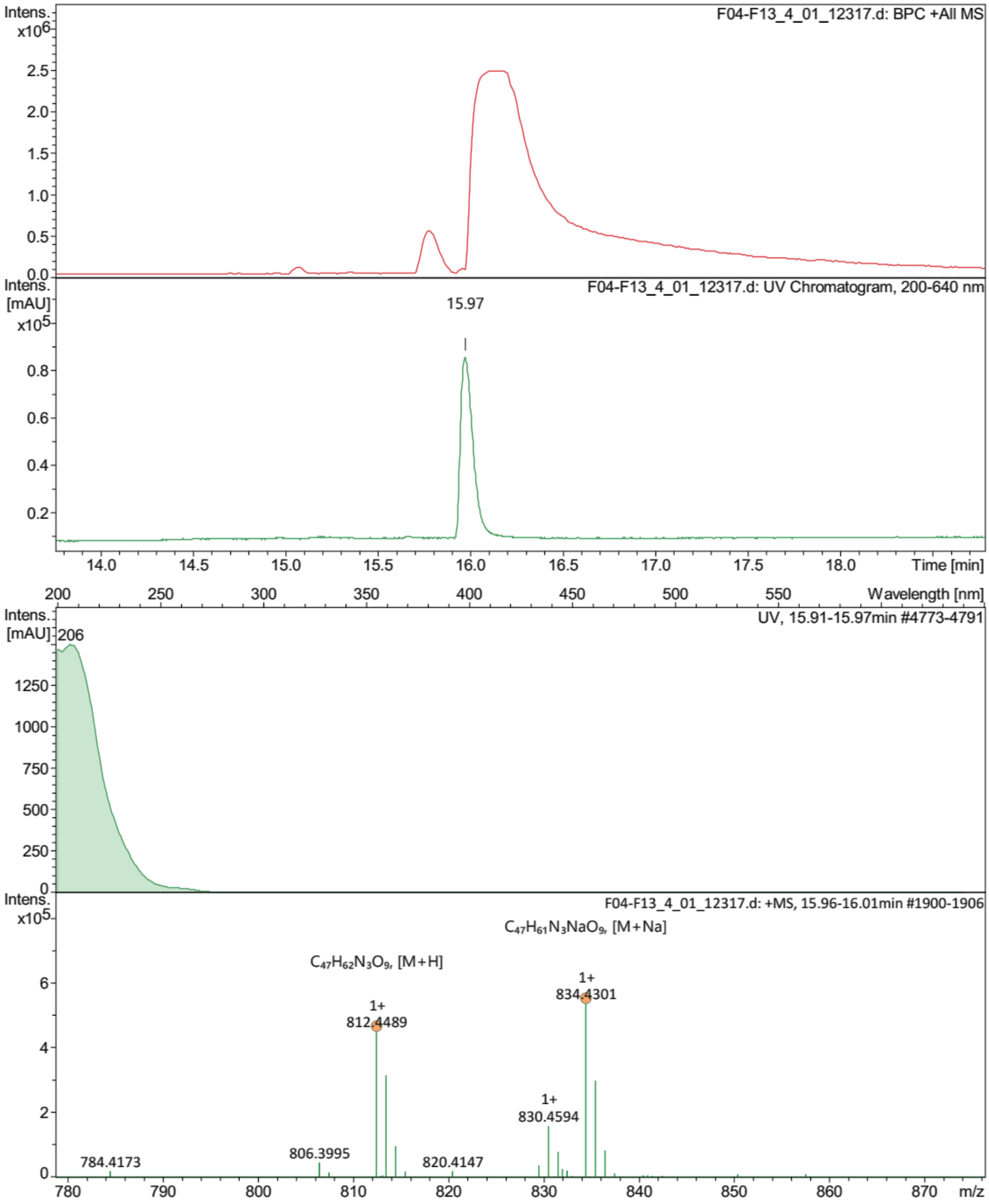

Bruker Compass DataAnalysis 4.4 printed: 14.07.2023 10:56:41 by: sel22 Page 1 of 1

Figure S28. HRESIMS spectrum of **5**.

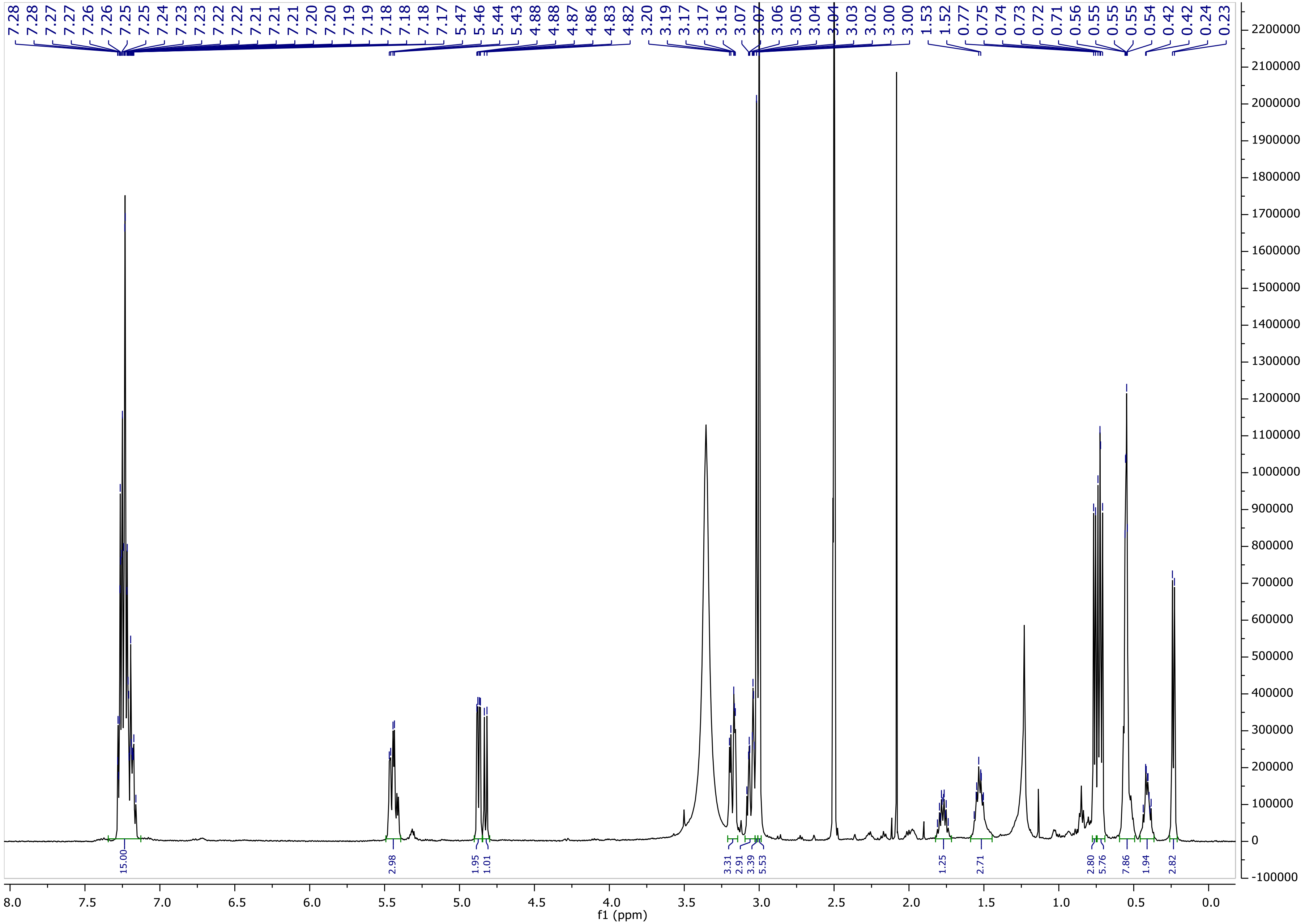

Figure S29. ^{1}H NMR spectrum of **5** in DMSO-d_6 at 500 MHz.

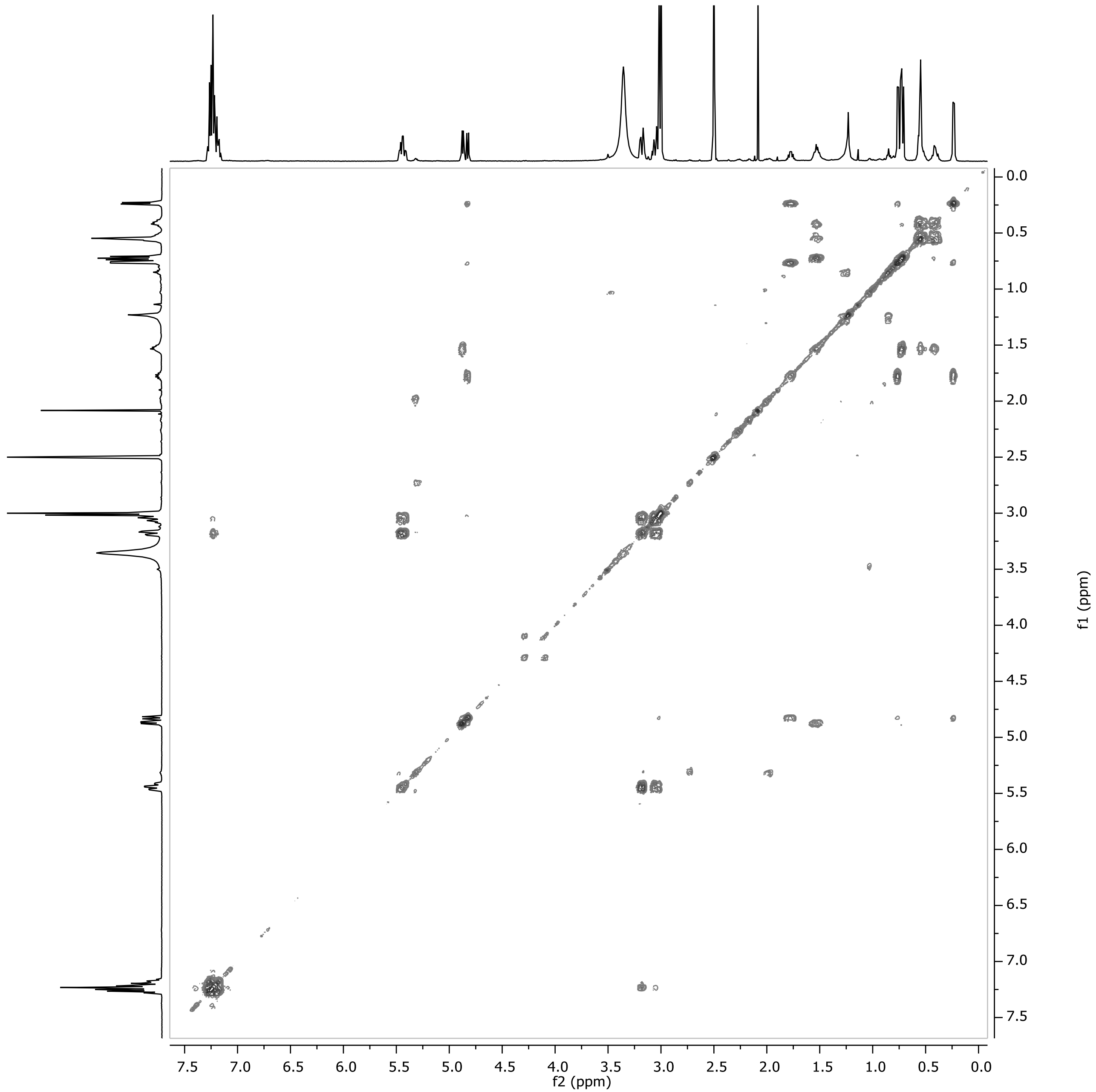

Figure S30. ^{1}H-^{1}H COSY spectrum of **5** in DMSO-d_6 at 500 MHz.

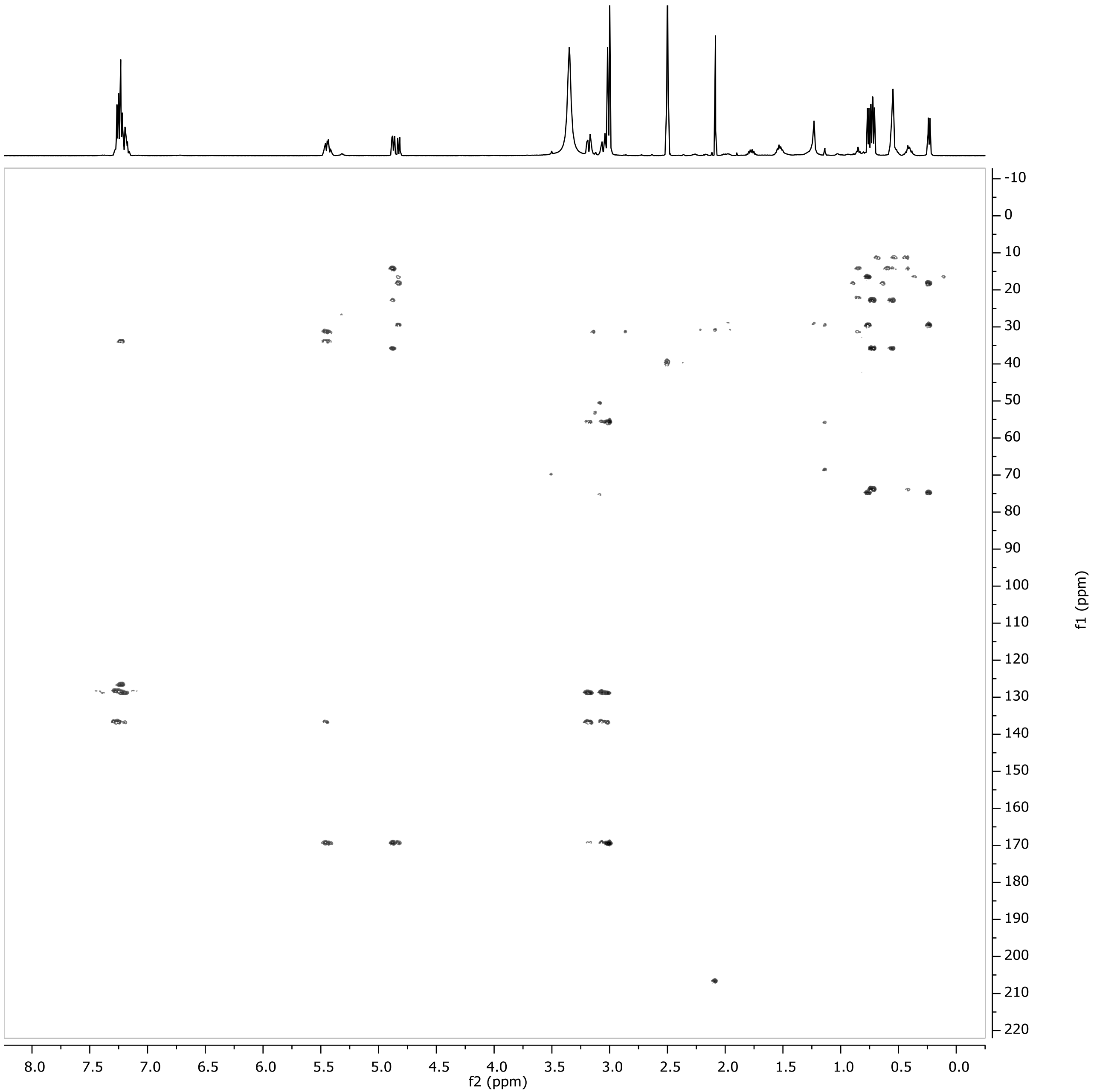

Figure S31. HMBC spectrum of **5** in DMSO-d_6 at 500 MHz.

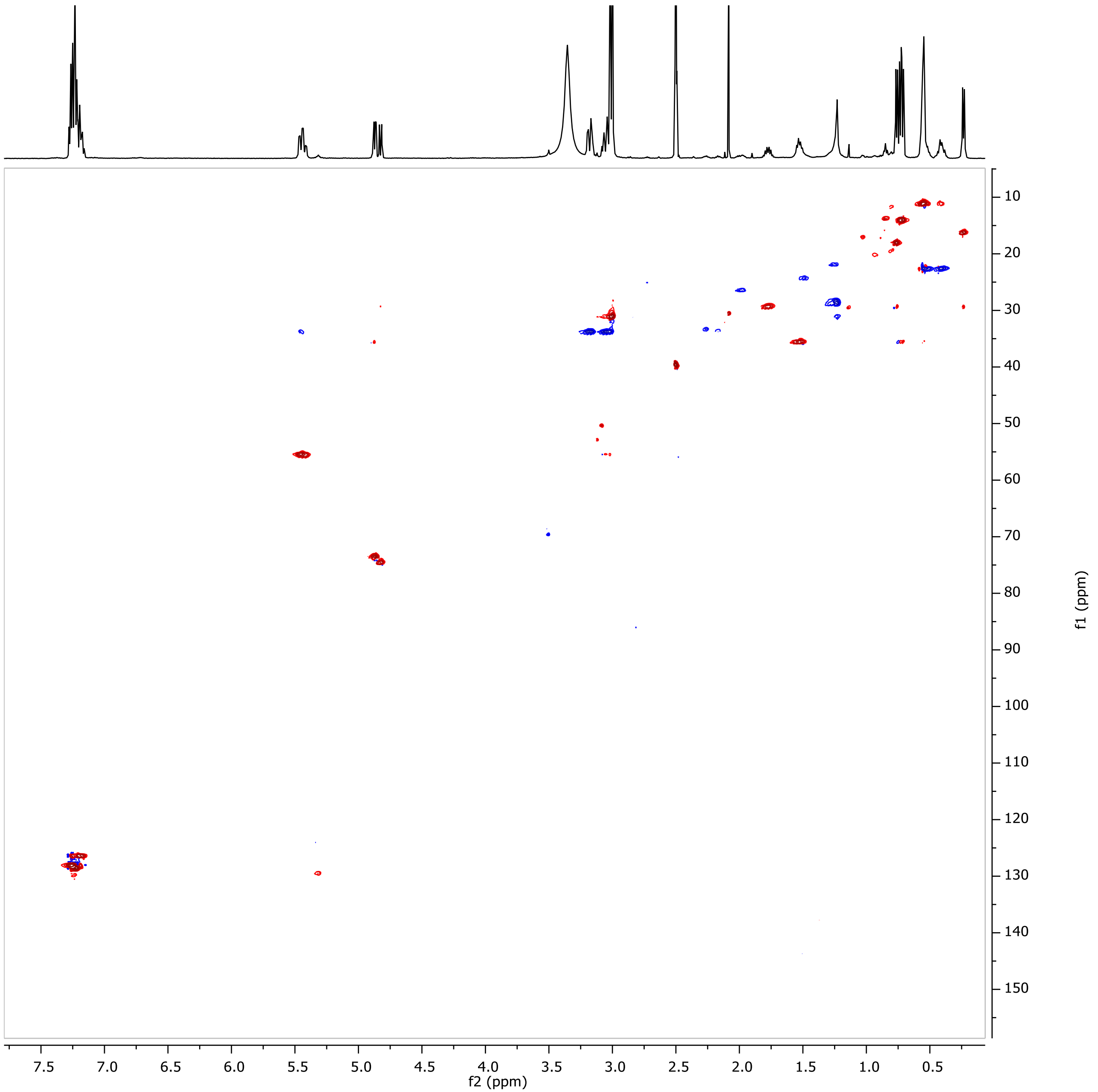

Figure S32. HSQC spectrum of **5** in DMSO-d_6 at 500 MHz.

Generic Display Report

Analysis Info

		Acquisition Date	08.08.2023 02:51:32
Analysis Name	S:\DATA\MaXis\rto20_Rita Toshe\23_08\MY07955-Rice-S1-F03-F13_43_01_12784.d		
Method	pos_säure_10000_screening_ms_100_2500_line.m	Operator	ate06
Sample Name	MY07955-Rice-S1-F03-F13	Instrument	maXis
Comment	Screening01<br>Waters Acquity UPLC BEH C_18 1,7um 2.1x50mm		

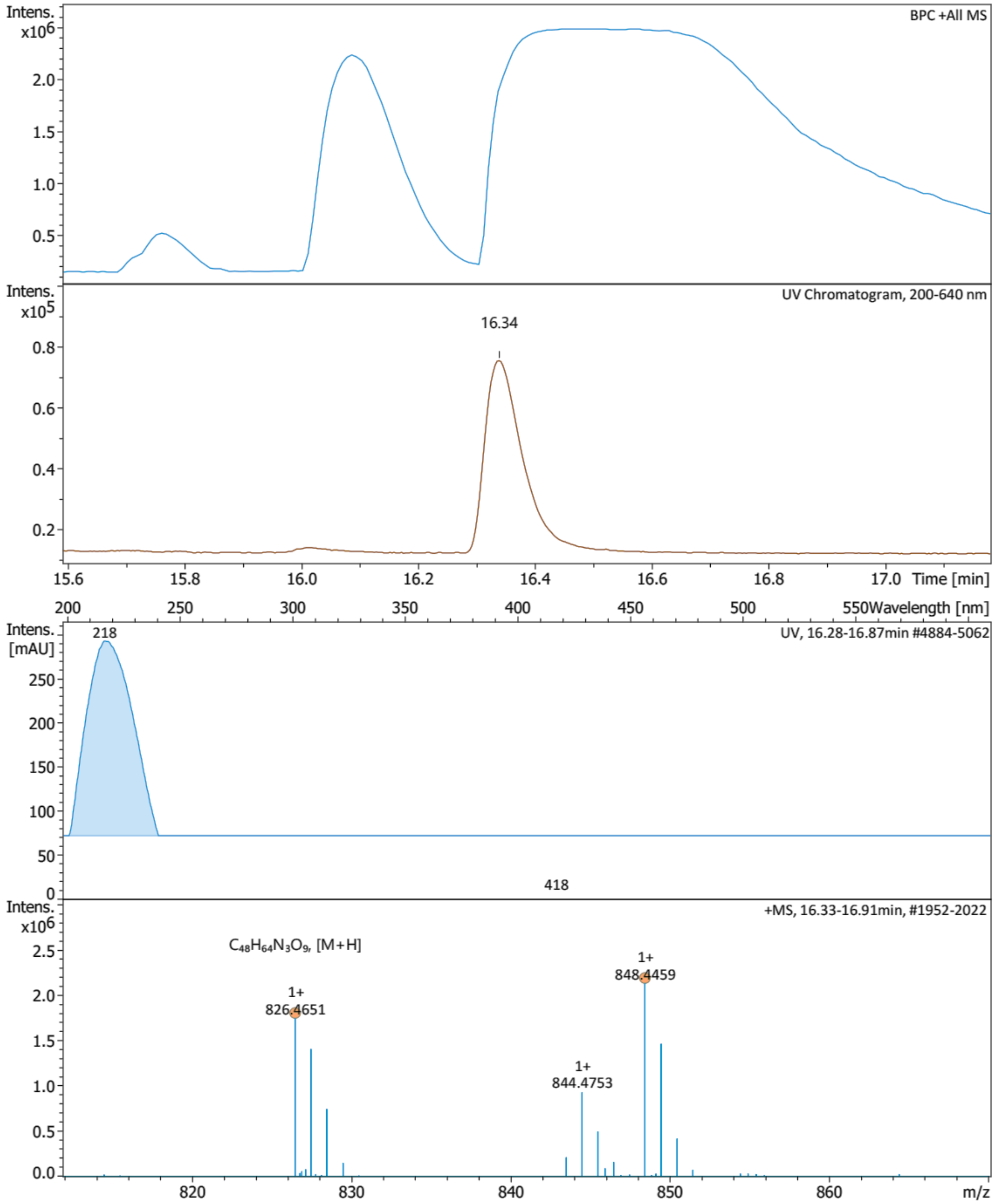

Bruker Compass DataAnalysis 6.1 printed: 25.10.2023 15:39:51 by: sel22 Page 1 of 1

Figure S33. HRESIMS spectrum of **6**.

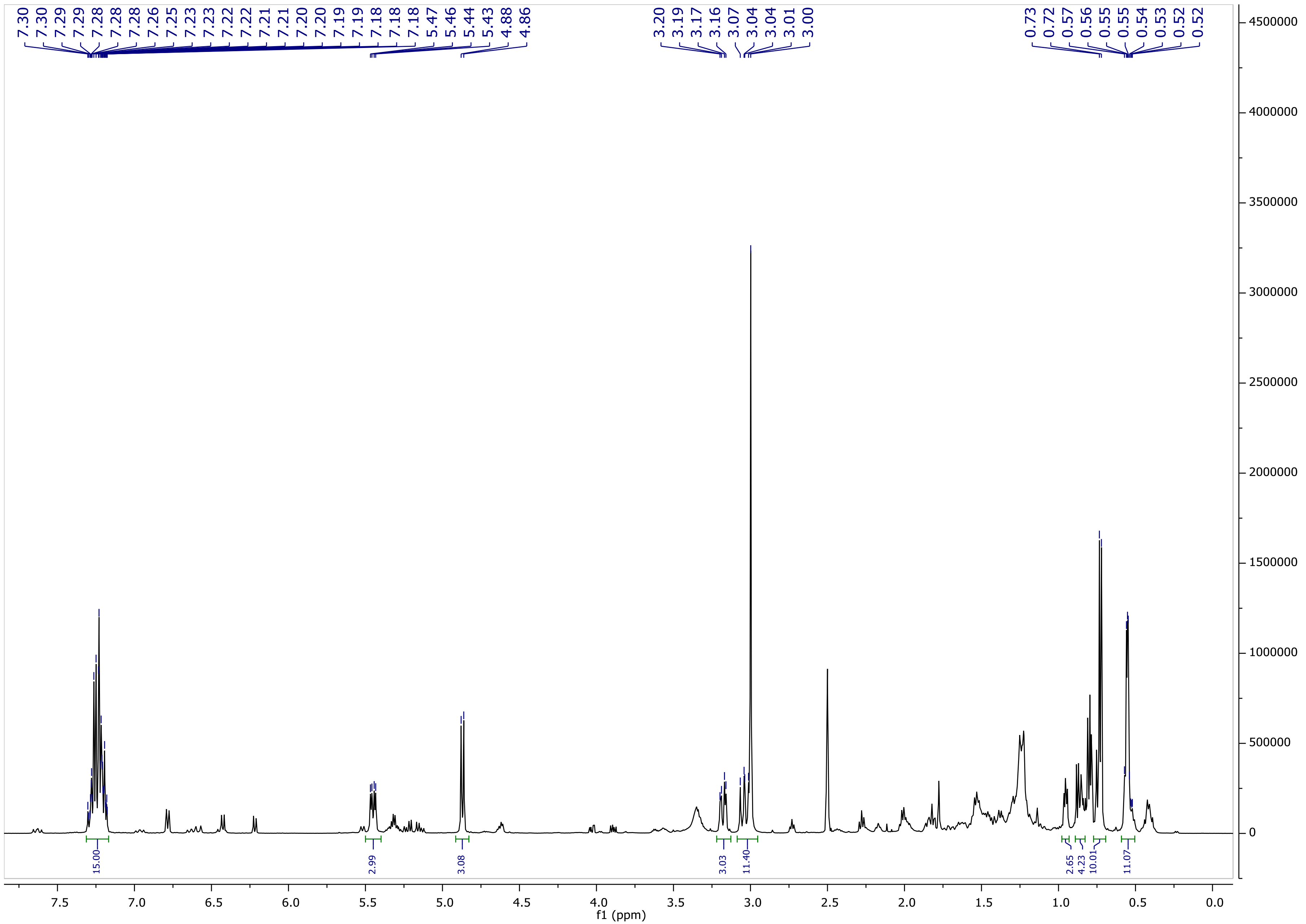

Figure S34. ^{1}H NMR spectrum of **6** in DMSO-d_6 at 500 MHz.
